# Genetic survey of crucian carp *Carassius carassius* populations in Hungary for a conservation project to establish live gene bank

**DOI:** 10.1038/s41598-025-93468-6

**Published:** 2025-03-14

**Authors:** István Lehoczky, Fatema Ali Al Fatle, Quynh Nguyen Thi, Erika Edviné Meleg, Zoltán Sallai, Gergely Szabó, Gábor Fekete, István Kópor, Eszter Várkonyi, Dániel Péter, Gábor Beliczky, Balázs Kovács, Béla Urbányi, Tamás Molnár

**Affiliations:** 1 Institute of Aquaculture and Environmental Safety, Hungarian University of Agriculture and Life Sciences, Gödöllő - Keszthely, 2100 Hungary; 2 Department of Biology, College of Science, University of Baghdad, Baghdad, Iraq; 3 Jabiru Vietnam Company Limited, Van Giang, Hung Yen Vietnam; 4 Vaskos Csabak Ltd., Szarvas, 5540 Hungary; 5 Institute for Farm Animal Gene Conservation, National Centre for Biodiversity and Gene Conservation, Gödöllő, 2100 Hungary

**Keywords:** *Carassius carassius*, Hybridisation, Microsatellite, Migration, Mitochondrial DNA, Population structure, Genetics, Genetic markers, Population genetics, Ecology, Biodiversity, Invasive species

## Abstract

The crucian carp (*Carassius carassius* Linnaeus, 1758) is a declining native European cyprinid, inhabiting small water bodies, primariliy threatened by climate change, anthropogenic impacts and invasive relative the Prussian carp. Despite conservation efforts across Europe, data on Carphatian Basin populations remain scarce. This study analyzed nine natural populations (257 individuals) in Hungary using thirteen microsatellite markers and mitochondrial DNA COI sequencing (187 individuals). Sequencing of mitochondrial DNA revealed a presumably introduced Baltic stock in addition to the Danube lineage and the presence of Prussian carp hybrids in part of the populations. Microsatellite markers also confirmed the latter, but there were populations in the southern region free of hybrids. Genetic diversity was found to be moderate (Ho: 0.49–0.61; Ar: 6.01–7.98). Depending on the genetic structure analysis method, two or three main units with low to moderate differentiation were detected (FST: 0.054–0.192). Based on gene flow, the Danube-Drava region showed a separation from the northern areas and the populations on the eastern bank of the Danube. Eight of the nine populations examined, especially the south Danube populations, could provide a good basis for the establishment of a genetically controlled gene bank of remaining crucian carp stocks, without hybrids.

## Introduction

Freshwater ecosystems cover less than 1% of the Earth’s surface, yet they serve as major biodiversity hotspots and provide vital ecosystem services^1^. Ponds, isolated rivers, and oxbows provide a habitat for many specialist and rare species absent from other water bodies^2^, thus playing a crucial role in maintaining biodiversity in fragmented landscapes^3^. Crucian carp (*Carassius carassius* Linnaeus, 1758) is one of the rare native cyprinid species in Central and Eastern Europe, preferring these habitats^4^. The species can survive extreme high summer temperatures and extreme low oxygen levels under ice cover in its habitat^5^. However, it is characterized as a weak competitor. It is usually absent from waters with rich ichthyofauna and abundant predatory species but can be present in high abundance in waters without other or a very limited number of fish species^6^. The species is an important inhabitant of periodically flooded ponds with oxygen deficiency^7–9^ and plays a vital ecological role in sediment-clogged ponds as a food source for predatory fishes^10–12^. However, populations of the species are declining in European waters. For example, a 72% decline in crucian carp distribution was observed between the 1950s–1980s and the 2010s in the UK^8^, also in the Danube drainage system for unknown reasons. However, the most probable reason for the decline of *C. carassius* populations is competition with the alien *Carassius gibelio* Bloch, 1782 (Prussian carp) in non-optimal habitats^6^. Based on the literature , the main reasons for the decline of the species are the loss and degradation of habitats, limited food supply, competition with *C. gibelio* and *C. auratus* (goldfish), and hybridization with individuals of the same species (13). In some European countries (i.e.: Czech Republic), *C. carassius* became critically endangered (14). Possible ecological mechanisms of the rapid decline of the crucian carp attributable to resource competition with the *C. gibelio* were identified^8,9,12,15–17^. Prussian carp is an invasive species with a unique strategy. In some cases, the population is composed of purely triploid females that spawn with crucian carp males and use their sperm to induce egg cell division, resulting in clones of offspring (gynogenetic reproduction)^17^. However, in some rare cases, paternal genes can also be transmitted to the offspring^18^. In other cases, the Prussian carp is present in a diploid form and hybridizes with crucian carp and other cyprinid species. The hybrid offspring compete with the original species for resources^19^. The hybrids are fertile and can backcross with the parental species^20,21^. Prussian carp was introduced to Europe from Asia, where it became very common^22,23^. The species is also widespread in Hungary^21,24^. Demeny et al.^25^ proved in pond experiments that the presence of Prussian carp has a strong negative effect on crucian carp. They found that juvenile crucian carp showed decreased numbers and lower growth rates when kept together with Prussian carp juveniles. The situation of crucian carp in Europe makes it necessary to start conservation and restoration projects. Experiences from successful restoration and conservation projects in Europe showed that the species can survive and prosper in shallow, small-sized ponds where predatory species, otters, anglers, and most importantly, Prussian carp are absent^5,8,9,26,27^. Before the extensive river regulation programs conducted in the latter part of the nineteenth century, the Crucian carp was widespread and common in Hungary. According to the results of fish fauna surveys, its populations have now become rare and have disappeared in many locations. The species is currently classified as one of the non-catchable species from an angling perspective; thus, declaring it protected would be justified^28^.

To establish successful conservation and restoration projects, the genetic background of the species or population in focus must be clarified^29^. For this reason, a molecular genetic protocol was developed in the UK to identify pure-bred crucian carp, goldfish, and common carp and reliably distinguish them from their hybrids and backcrosses of at least the first two generations^30^. They developed and applied a set of 5 microsatellite markers with diagnostic allele size ranges for all three species. Hybrids between all three species were present in the samples. Differentiation between some categories of hybrid (e.g.: goldfish x crucian carp, crucian carp x common carp, etc.) based on morphological grounds proved to be unreliable. Janson et al.^31^ used nine microsatellite DNA markers to describe the genetic variability of Swedish crucian carp populations in the wild. They examined 234 individuals from 20 locations in various parts of Sweden. The genetic distances of crucian carp populations indicated that southern populations shared a common history, together with a pond population in the province Småland. It is suggested based on population genetic distances that populations in the further northern parts of Sweden share a much more complex history of crucian carp distributions. They found some ponds with potentially old populations without admixture but described also several ponds stocked with fish from many sources.

Similarly to the experiences in the UK^8^ and the Czech Republic Czech^13^, where stocking and restocking programs were initiated alongside tailored conservation management of habitats, it has become necessary to establish a conservation program for crucian carp in Hungary. This program will initially focus on ex-situ conservation of the species for the purpose of stocking and restocking suitable habitats. Subsequently, in-situ conservation efforts will also be implemented.

Up to now, information on the genetic background of the species in Hungary was very limited. The present study describes the genetic variability and purity of Hungarian crucian carp populations to establish a well-grounded conservation program.

## Material and methods

### Sampling and DNA extraction

**Fig. 1:**
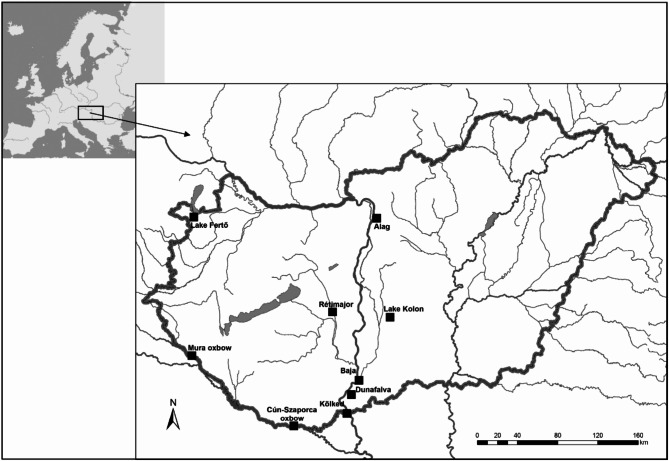
Map of sample locations in Hungary.

Fin tissue samples were collected from 257 individuals representing nine different natural populations (Table S1, Fig. 1). The samples were collected from live fish in the nature. The fish were collected with an electric fishing machine (Samus 725 MP ), and a 1 mm × 1 mm fin tail piece was collected (fin clipping) from them in the moments after the catch (maximum duration of 1 min) while the fish were still under the influence of the electric shock. After disinfecting the small wound surface, the fish were released into their natural environment. The fin tissue samples were kept in 2 ml Eppendorf tubes containing absolute ethanol (AnalaR NORMAPUR ACS, VWR), then subsequently labelled and transported to the molecular laboratory located at the National Centre for Biodiversity and Gene Conservation (NBGK-HGI, Hungary). At the institute, the samples were stored at a temperature of − 20 °C.

Total genomic DNA was extracted from fin clips using the Qiagen DNeasy® Blood and Tissue Kit (Qiagen GmbH, Hilden, Germany) according to the manufacturer’s instructions. A spectrophotometer (NanoDrop 2000c, Thermo Fisher Scientific) was used to assess the quality and concentration of extracted DNA.

### Amplification and sequencing of the mitochondrial COI region

Using the universal primer pairs CO1-FF2d-F (5′- TTCTCCACCAACCACAARGAYATYGG-3′) and CO1-FR1d-R (5′- CACCTCAGGGTGTCCGAARAAYCARAA-3′)^32^, the Cytochrome Oxidase C subunit I gene (COI) was amplified for sequencing analysis in 187 out of 257 samples collected from nine natural populations in Hungary. The amplification procedure consisted of the following steps: initial denaturation at 94 °C for 2 min, followed by 30 cycles of denaturation at 94 °C for 40 s, annealing at 52 °C for 1 min, extension at 72 °C for 1 min, and a final extension at 72 °C for 7 min. The final concentrations of the components in the 15 μl PCR reaction mixtures were as follows: Genomic DNA: 2 ng/μl; 10× DreamTaq Buffer: 0.8×; MgCl₂: 1.6 mM; dNTPs: 0.67 mM; Primers: 0.53 μM (for each primer); DreamTaq DNA Polymerase: 0.033 U/μl. The PCR products were purified using the NucleoSpin Gel and PCR Clean-up Kit (Macherey–Nagel, Düren, Germany). The purified products underwent sequencing using the Big Dye Terminator v. 3.1 Cycle Sequencing Kit (Applied Biosystems, Foster City, CA, USA) following the instructions provided by the manufacturer. An ABI 3130 genetic analyzer (Applied Biosystems, Foster City, CA, USA) was used to detect the sequences.

### Microsatellite analysis

Thirteen microsatellite markers originally developed for other cyprinid species as described before^33–38^ were cross-amplified in *C. carassius*. The PCR reactions were performed in a 15 μl reaction volume with the following final concentrations of the components in the reaction mixture: Genomic DNA: 2 ng/μl; 10× DreamTaq Buffer: 1.0×; MgCl₂: 2.0 mM; dNTPs: 0.67 mM; Tailed Forward Primer: 0.27 μM; Reverse Primer: 0.27 μM; Fluorescently-Labelled Tail Sequence: 0.067 μM; DreamTaq DNA Polymerase: 0.033 U/μl. The PCRs were carried out by the Kyratec PCR thermal cycler (Kyratec, Australia) using the following protocol: 3 min of initial denaturation at 94 °C, followed by 30 cycles of denaturation at 94 °C for 30 s, annealing temperature at 53–60 °C for 30 s, and extension at 72 °C for 30 s. The final extension lasted 5 min at 72 °C. The final concentrations of the components in the 13.4 μl PCR reaction mixture for the CypG24 marker were as follows: Genomic DNA: 2.24 ng/μl; Multiplex Mix: 1X; Tailed Forward Primer: 0.112 μM; Reverse Primer: 0.112 μM; Fluorescently Labelled Tail Sequence: 0.112 μM; Q Solution: 1X. The PCR reactions were programmed in a Kyratec PCR thermal cycler (Kyratec, Australia) as follows: initial denaturation at 95 °C for 10 min, followed by 30 cycles of 94 °C for 1 min, annealing temperature at 57 °C for 1 min, and 72 °C for 1 min. At 60 °C, the final extension was performed for 45 min. As mentioned, for PCR amplification, universal-tailed primers were utilized. An universal sequence (5'-ATTACCGCGGCTGCTGG-3) was linked to 5’ end of the forward primers^39,40^. The universal primer (tail) was labelled with different fluorescent dyes (NED, VIC, 6-FAM, and PET). After running at 90 mA for 1:20 min on a 1.5% agarose gel, the approximate sizes of the PCR products were estimated using a 100 bp ladder. For the precise analysis of microsatellite bands and allele sizes were used capillary electrophoresis system. The automated ABI Prism 3130 genetic analyser (Applied Biosystems, Foster City, CA, USA) was used for sizing the fluorescently labelled PCR products.

### Statistical analysis

MICRO-CHECKER version 2.2.3 (number of randomizations: 1000, 95% CI) was used to assess the presence of null alleles^41^. Allele number (Na), effective allele number (Neff), observed (Ho) and unbiased expected heterozygosity (uHe), fixation index (F), and the significance of the assumptions for Hardy–Weinberg Equilibrium were estimated using GenAlEx 6.5^42^ while allele richness (AR) and individual allele richness (ARp) were estimated using HP RARE 1.0^43^. Genetic diversity indices were compared in each subpopulation using the IBM SPSS 25 software package with the Kruskal–Wallis test (Bonferroni correction, significance threshold of 0.006). Genetic bottleneck, indicating potential population declines, was tested with Bottleneck 1.2.02 software^44^ under a two-phased mutation model (TPM) using default settings. The significance was estimated by the Wilcoxon sign-rank test with 1000 iterations. The effective population size (Ne) was estimated with LD and heterozygote excess methods implemented in NeEstimator 2.01 software^45^. Detection of a recent reduction in population size was performed with the M-ratio test^46^ implemented in Arlequin v.3.5.2.2^47^.

The analysis of the molecular variance (AMOVA), was performed using the GenAlEx 6.5 programme. Due to the presence of null alleles, the estimation of the pairwise Fst of Weir was performed with ENA correction, while the INA correction was used for Cavalli-Sforza and Edwards genetic distance, using FreeNA software^48^. For the computation of 95% confidence intervals, bootstrap was used with 10,000 replicates. Neighbor-Joining (NJ) tree was constructed using Populations 1.2.32 software based on Cavalli-Sforza and Edwards genetic distance and bootstrap on individuals with 10,000 repetition^49^, for visualisation MEGA11^50^ was used. To determine population structure, we used the Bayesian algorithm implemented in STRUCTURE software^51,52^. The most probable cluster number (K) was estimated by using both posterior probabilities (highest lnP(D)) and the ΔK method^53^, in the Structure Selector software^54^. To determine the cluster number, an admixture scenario with allele frequencies correlated was chosen, the burn-in was set to 10,000 and the number of further MCMC runs was set to 200,000. Calculations were repeated 10 times for each K. The STRUCTURE analysis was carried out in a hierarchical system in several steps. Discriminant analysis of principal components (DAPC) using microsatellite loci and populations was performed in the R environment (4.2.1) with the adegenet 2.1.1.7 package^55^. The paired Mantel test (9999 permutations), between pairwise Fst values and geographic distances among populations, was calculated in the GenAlEx 6.5 software.

The relative directional migration network was calculated by the method based on Jost’s D using divMigrate-online software^56^ and 1000 bootstrap iterations for the statistical testing of the asymmetry between migration rates of all population pairs.

## Results

### Mitochondrial DNA analysis

**Fig. 2:**
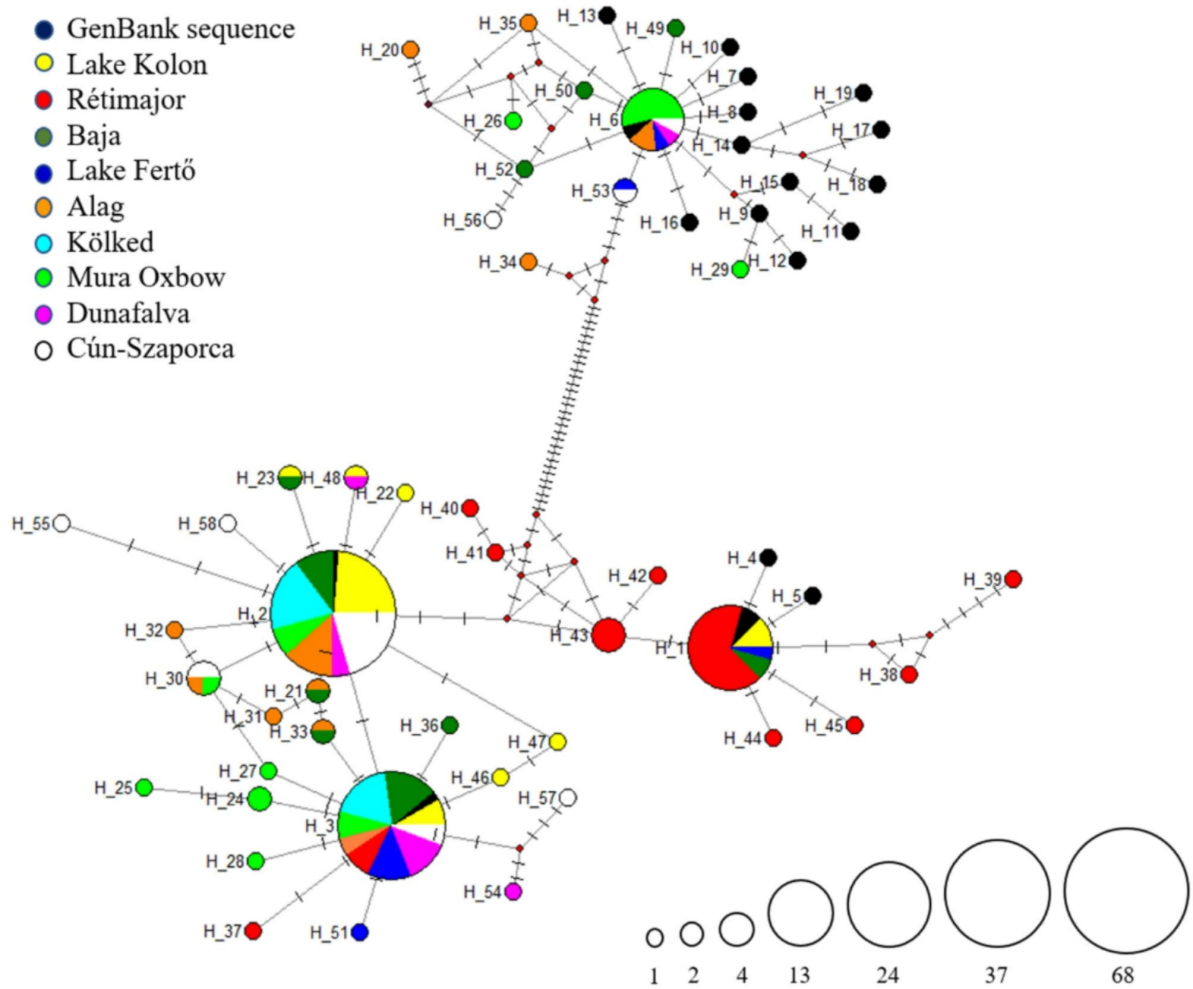
The haplotype network of the mitochondrial Cytochrome Oxidase C subunit I sequences in the nine crucian carp populations (GenBank sequences of *Carassius carassius* and *Carassius gibelio* included: HQ961040.1, HQ960942.1, HQ960610.1, MF135912.1, KJ128441.1, KJ128440.1, MW564549.1, MW564266.1, HM392055.1, HQ960965.1, HQ960884.1, HQ600705.1, MF036177.1, MF083603.1, KT716381.1, JQ319113.1, JQ979156.1, JQ979150.1, JQ979149.1, JQ979143.1) of Hungary. The size of the circles represents the number of haplotypes, based on the scale in the bottom right corner.

Based on the COI mtDNA sequences 58 haplotypes were identified. Three clusters can be distinguished based on the mitochondrial DNA sequence network (Fig. 2). The first cluster contains the two dominant haplotypes that were found to correspond to the genomic sequences of *C. carassius*: H2 was found to match the sequence of the GenBank collected in the Stibůrkovská jezera nature reserve in the Czech Republic (HQ960942.1), while the haplotype H3 was identical to a sequence from a collection (HQ960610.1) also from Žebětín Lake near Brno, Czech Republic. Within the second *C. carassius* cluster, the dominant haplotype was the H1, identical to KJ128440.1 GenBank sequence from Sweden and contained only one nucleotide difference compared to haplotypes H4 and H5, these are GenBank sequences from the Baltic region (MF135912.1 and KJ128441.1), collected at Nemunas River, Lithuania and Sweden, respectively. The third cluster, dominated by the H6 haplotype, showed species-level segregation based on GenBank sequences, so that the haplotypes classified belonged to the species *C. gibelio*. The latter individuals were presumably hybrid individuals in the Hungarian stocks, as they phenotypically corresponded to the crucian carp.

There were large differences between the populations according to the genetic diversity of the COI sequence. Higher diversity was identified in the Lake Fertő, Alag, and Mura Oxbow, since Kölked, Lake Kolon showed low diversity levels (Table 1).

**Table 1:** Genetic diversity data of the mitochondrial COI sequences in the nine crucian carp populations of Hungary.

Stock	N	Nh	S	Eta	K	Hd (mean ± SD)	Pi (mean ± SD)
Lake Kolon	27	8	10	10	1.6	0.641 ± 0.099	0.00270 ± 0.00076
Rétimajor	30	11	19	22	3.0	0.738 ± 0.080	0.00493 ± 0.00108
Baja	19	7	9	9	1.9	0.784 ± 0.067	0.00313 ± 0.00089
Lake Fertő	12	8	45	46	22.0	0.848 ± 0.104	0.03604 ± 0.00478
Alag	21	11	43	46	14.7	0.819 ± 0.082	0.02487 ± 0.00628
Kölked	20	2	1	1	0.47	0.479 ± 0.072	0.00078 ± 0.00012
Mura Oxbow	23	10	48	50	20.8	0.862 ± 0.002	0.03407 ± 0.00346
Dunafalva	11	5	45	45	8.5	0.764 ± 0.107	0.01394 ± 0.00909
Cun-Szaporca	24	9	48	49	9.9	0.663 ± 0.107	0.01624 ± 0.00638

*N* number of sequences, *Nh* number of haplotypes, *S* number of polymorphic (segregating) sites, *Eta* total number of mutations, *K* average number of nucleotide differences, *Hd* haplotype diversity, *Pi* nucleotide diversity, *SD* Standard Deviation.

Fu’s F_S_ was not significant in any of the populations, while Tajima’s D test of selective neutrality was only significant in the Dunafalva population. This showed a negative value, indicating an excess of rare nucleotide variants, and a population size expansion after a recent bottleneck. The Chakraborty test showed a significant difference between the expected and observed haplotype numbers in three populations (Lake Kolon, Rétimajor, and Cún-Szaporca) indicating population amalgamation (Table S2).

### Microsatellite analysis

#### Genetic diversity and population size

Since both the mitochondrial DNA of the Rétimajor stock and the microsatellite (structure, DAPC) analysis (Figs. S1, S2) indicated a non-Hungarian origin, it was excluded from the microsatellite analysis. For the remaining eight populations, the markers showed different levels of null alleles. No null alleles were detected for Dunafalva, Kölked, and Baja. For the Cún-Szaporca, null alleles were detected at three loci (MFW7, HLJYJ028, HLJYJ046), and for the Lake Fertő, four (Gf1, Gf29, HLJYJ028, HLJYJ046), three (Gf1, Gf29, HLJYJ046) for the Lake Kolon, two (HLJYJ028, HLJYJ046) for the Alag population and five (Gf1, HLJYJ017, HLJYJ028, HLJYJ046, HLJYJ041) for the Mura Oxbow population. The following loci were monomorphic: GF1 in the Baja population, HLJYJ082 in the Dunafalva population, and both GF1 and HLJYJ082 in the Kölked population. The loci Gf29, MFW7, J62, HLJYJ029, HLJYJ028, HLJYJ046 in the Alag population, Gf29, MFW7, J62 in the Baja population, J62 in the Kölked population, Gf1, Gf29, MFW7, HLJYJ028 in the Lake Kolon population, Gf1, Gf29, MFW7, J62, HLJYJ029 in the Dunafalva population, Gf1, Gf29, MFW7, HLJYJ028, HLJYJ029 in the Fertő population, Gf1, YJ10, Gf29, YJ0022, HLJYJ028, HLJYJ029, HLJYJ046 in the Mura Oxbow population and MFW7, HLJYJ028, HLJYJ046 in the Cún-Szaporca population were all found to be out of the HW equilibrium.

None of the genetic diversity parameters showed significant differences among the populations studied. The highest allelic diversity was detected in the Mura Oxbow and Drava River (Cún-Szaporca) systems. Low estimated effective population size was found in the Alag and the Cún-Szaporca populations (Table 2). The BOTTLENECK’s Wilcoxon signed-rank test for heterozygosity excess or deficiency did not show evidence of a recent population grow, or bottleneck in any of the populations. All populations showed normal L-shaped distribution of allelic frequencies (Table 2). In contrast, the Garza-Williamson M-ratio test resulted in low values (below 0.68) in all of the natural populations indicating a recent reduction in the effective size of the populations (Table 2, Table S3).

**Table 2:** Mean genetic diversity parameters and the estimated effective population size of eight natural crucian carp populations of Hungary based on microsatellite data.

Population/parameter	Lake Kolon	Baja	Lake Fertő	Alag	Kölked	Mura-Oxbow	Dunafalva	Cún-Szaporca
Na	8.15 ± 7.27	8.15 ± 7.67	9.00 ± 7.29	8.30 ± 5.54	7.23 ± 7.19	9.46 ± 8.16	7.23 ± 6.94	10.23 ± 9.19
Neff	4.57 ± 4.14	5.00 ± 5.03	5.41 ± 5.49	5.12 ± 4.25	4.72 ± 5.09	6.07 ± 6.33	5.13 ± 5.55	6.14 ± 6.85
Ho	0.55 ± 0.30	0.57 ± 0.35	0.53 ± 0.24	0.61 ± 0.24	0.49 ± 0.37	0.56 ± 0.26	0.51 ± 0.35	0.53 ± 0.31
uHe	0.61 ± 0.27	0.55 ± 0.34	0.62 ± 0.26	0.66 ± 0.26	0.50 ± 0.37	0.65 ± 0.26	0.54 ± 0.34	0.59 ± 0.31
F	0.069 ± 0.36	−0.081 ± 0.24	0.078 ± 0.31	0.018 ± 0.23	0.013 ± 0.11	0.087 ± 0.33	0.099 ± 0.32	0.092 ± 0.15
AR	6.34 ± 4.92	6.64 ± 5.65	7.69 ± 5.94	6.99 ± 4.34	6.01 ± 5.41	7.98 ± 6.12	6.75 ± 6.24	7.74 ± 6.43
ARp	0.22 ± 0.45	0.60 ± 1.20	0.59 ± 0.91	0.75 ± 0.84	0.16 ± 0.42	0.55 ± 0.82	0.46 ± 0.99	0.46 ± 0.50
Ne (95%Cl)	138.9 (76.0–563.7)	inf. (235.0-inf.)	200.6 (73.5-inf.)	26.4 (20.9–34.5)	192.0 (79.4-inf.)	485.6 (112.8-inf.)	inf. (140.9-inf.)	59.7 (44.8–86.7)
G-W M	0.28 ± 0.20	0.29 ± 0.16	0.29 ± 0.19	0.28 ± 0.19	0.31 ± 0.17	0.27 ± 0.17	0.32 ± 0.20	0.30 ± 0.19
T.P.M	0.786	0.791	0.684	0.946	0.700	0.839	0.909	0.305

*Na*  number of alleles, *Neff* number of effective alleles, *Ho* observed heterozygosity, *uHe* unbiased expected heterozygosity, *F* inbreeding coefficient, *AR* allelic richness, *Arp* private allelic richness, *Ne* effective population size, *inf.* Infinite, *G–W M* Garza–Williamson M-ratio test (mean ± SD), *T.P.M.* significance of Wilcoxon signed rank test for heterozygote excess (He) based on T.P.M. model.

Microsatellite loci Gf1 and Gf29 fragment sizes^30^ indicated varying levels of hybridisation with *C. gibelio* in the natural populations. Kölked population was pure *C. carassius* (0/29), whereas the Alag population contained only hybrids (30/30). The remaining populations contained the following hybrid ratios: Dunafalva 1/20, Cún-Szaporca 5/31, Mura Oxbow 16/25, Lake Kolon 17/33, Lake Fertő 17/25, Baja 27/28 (Table S4).

#### Genetic differentiation and population structure

**Fig. 3:**
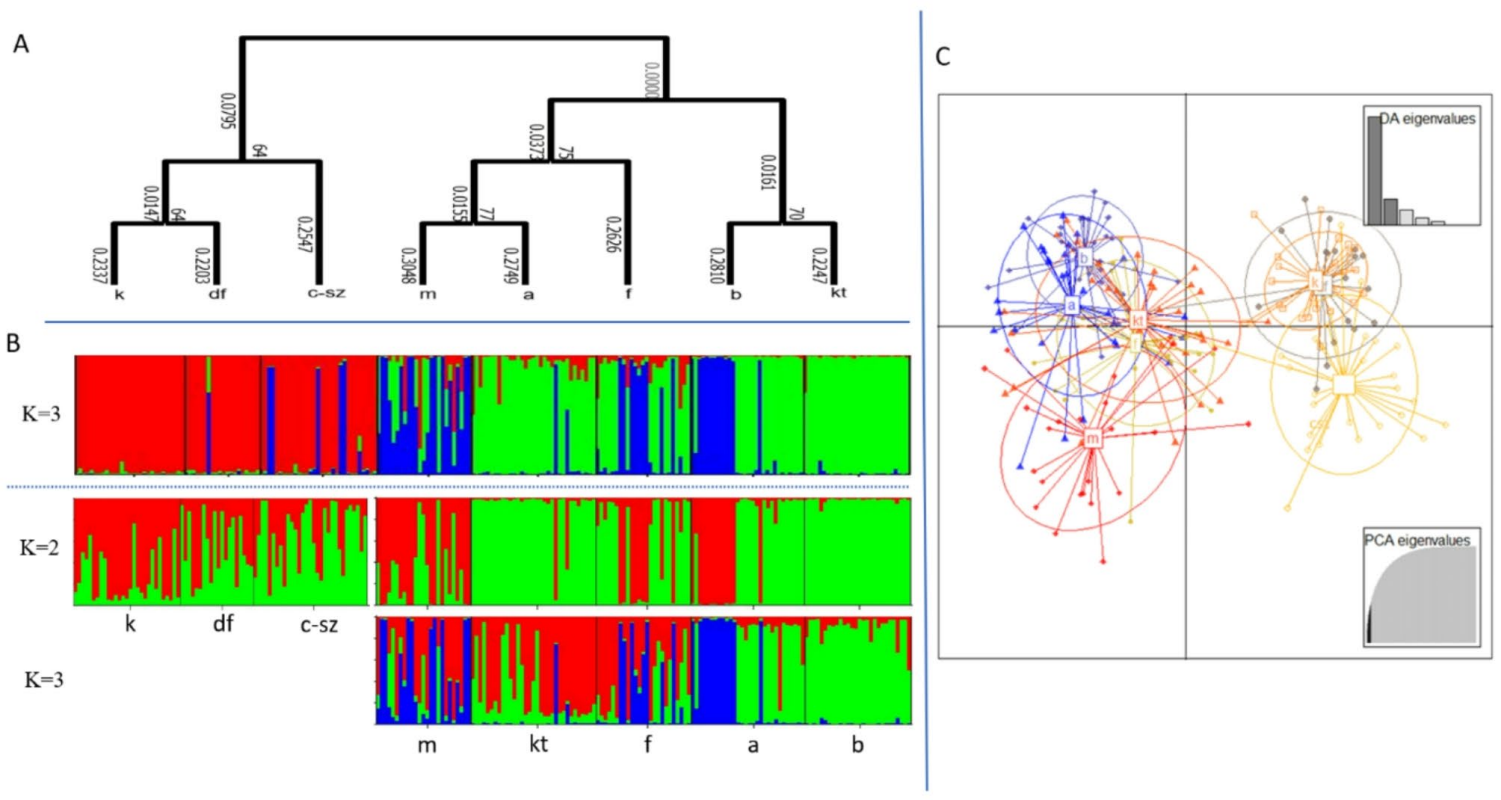
The genetic structure of the eight natural crucian carp populations of Hungary based on the Neighbor-joining tree (**A**), Hierarchical STRUCTURE analysis first for K = 3 (above dotted line) and for K = 2 and K = 3 (below dotted line) in the substructure (**B**), and DAPC analysis (C) performed on the microsatellite data. Populations: a—Alag, b—Baja, df—Dunafalva, f—Lake Fertő, c-sz—Cún-Szaporca, k—Kölked, kt—Lake Kolon, m—Mura Oxbow.

The global FST after ENA correction was 0.107 (CI 95%: 0.047–0.193). Within the eight natural populations, two large groups could be distinguished, confirmed by both FST, genetic distance values (Table S5) and DAPC analysis (Fig. 3C). Although the phylogenetic tree and the STRUCTURE analysis confirmed three groups (most probable K = 3, ΔK method), proving one of the groups defined by the previous methods and classifying the other into two additional groups. (Fig. 3A, B, Fig. S3). After running STRUCTURE analysis again in the three populations (k, df, and c-sz, marked with the red cluster), which appeared homogeneous, we obtained K = 2 clusters using both lnP(D) and ΔK methods. These were present in all three populations, although the Kölked population was dominated by one and the Cún-Szaporca population by the other cluster (Fig. 3B, Fig. S4). Re-analysing the remaining five populations resulted in two clusters using the the ΔK method and three clusters using the lnP(D) method. The two-cluster subdivision corresponds to the pattern characteristic of the populations within the first run, while the third cluster in the K = 3 subdivision is characteristic of the Lake Kolon population. (Fig. 3B, Fig. S5).

**Fig. 4:**
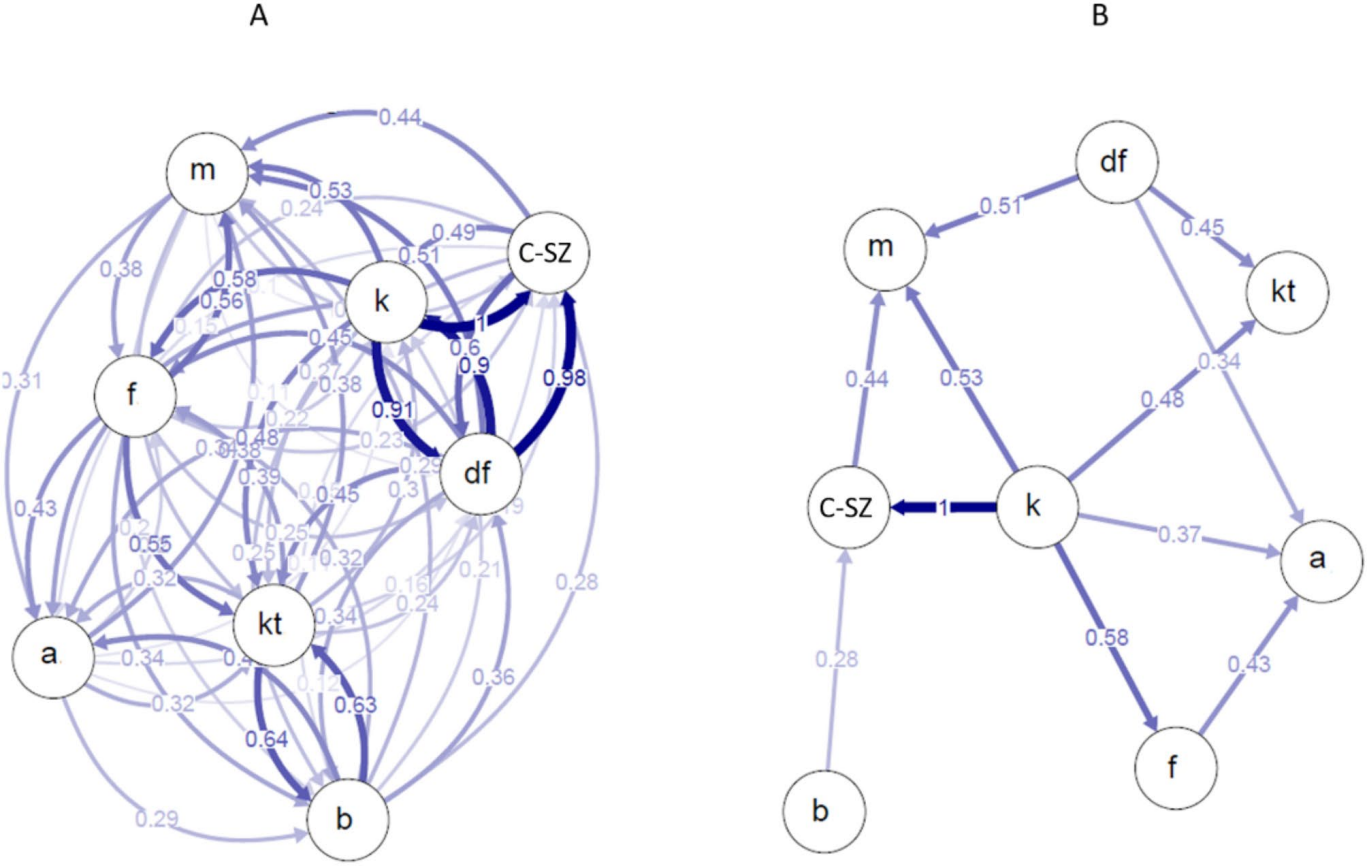
Directional relative migration estimated among eighth Hungarian crucian carp populations by divMigrate-online based on microsatellite data (D method): (**A**) all populations, no filter was used, (**B**): asymmetric migration, 1000 bootstrapping, no filter was used. The differently bolded arrows show the direction and strength of migration between populations. A bolder arrow indicates stronger migration. Populations: a—Alag, b—Baja, df—Dunafalva, f—Lake Fertő, c-sz—Cún-Szaporca, k—Kölked, kt—Lake Kolon, m—Mura Oxbow.

The calculated relative directional migration matrix (Fig. 4A) showed significant gene flow within the South Danube cluster (Dunafalva, Cún-Szaporca, Kölked) and between the Baja Lake Kolon populations on the other side of the Danube, but with less flow between areas on different sides of the Danube. Bootstrapping confirmed that statistically significant asymmetric migration occurs in several population pairs: the Kölked and Dunafalva populations act as source populations, while the Alag and Mura Oxbow populations show a directional migration towards them (Fig. 4B, Table S6).

## Discussion

### Mitochondrial DNA analysis-hybridisation

Jeffries et al.^57^ report on two lineages with distinct geographic distributions of the *C. carassius*, one in Northern and Eastern Europe and the other in the River Danube catchment using cytochrome b (cytb) gene sequence. Their suggestion is to consider the two lineages as separate conservation units. Although we used COI sequence, we obtained similar results based on mitochondrial DNA. The two major haplotypes of the Carpathian Basin population matched with two Czech sequences from GenBank, confirming the River Danube catchment lineage. The third haplotype, which was present only in the Rétimajor population, was matched with Baltic samples based on the sequence representing the other lineage typical in Northern Europe. The Chakraborty test indicated population amalgamation in three of the examined populations in our study. This seems to be confirmed in the Rétimajor population, as the local population was probably crossed with a population of Polish origin. The introduction of Polish individuals to the Hungarian habitats is inconsistent with the management recommendation suggested by Jeffries et al.^57^, which should be considered during the establishment of gene bank stocks. The explanation of other population amalgamations may be a mixing of natural, and local (in the case of Cún-Szaporca the Dravidian and Danubian) populations. Notwithstanding the above, anthropogenic influence (introductions from the Carpathian Basin) is also possible, as crucian carp have a very low dispersal propensity and a strong preference for lentic and isolated habitats^57^, so the potential for natural mixing of distant local populations is low.

The third mtDNA haplotype group in our study represented *C. gibelio* hybrids. The main cause of the decline/disappearance of the East-Central European populations is the emergence of Prussian carp due to interspecific competition^58,59^ and hybridization/introgression. In Hungary, where the Prussian carp appeared early, in the 1950s^18^, most of the natural crucian carp habitats are now invaded by the species. Our analysis identified hybrid individuals with different molecular markers at different levels. Of the individuals detected by mitochondrial DNA, seven were not confirmed by microsatellites, while microsatellite markers indicated significantly more hybrids, mainly based on the GF1 marker. In the original publication by Hanfling et al.^12^, this marker indicated a private allele of 298 bp in length in *C. carassius*, whereas it was longer (300–312 bp) in *C. gibelio*. In our study, 90 of the individuals identified as hybrids with this marker contained the 300 bp long allele in homozygous form, 11 individuals in heterozygous form with the 298 bp allele, and two individuals in heterozygous form with the 302 bp allele. This marker suggests that *C. gibelio* hybrids are widely distributed in the Hungarian area. However, the ratio measured in the GF1 marker is not fully confirmed by the GF27 marker and mtDNA data. Moreover, hybrids identified by the GF27 marker contained the alleles specific for *C. gibelio* in heterozygous form in all but three cases. It is conceivable that the original population studied by Hanfling et al.^12^ and the Carpathian Basin population differ significantly and the allele size for the GF1 marker cannot be extrapolated to Hungarian populations. Nevertheless, the GF27 marker and mtDNA data point to a significant degree of hybridization. Whether they are F1 hybrids, however, cannot be determined from the characteristics of the two microsatellite markers. The homozygote genotype of the microsatellite alleles above indicates second or later hybrid generations. It would be worthwhile to clarify the extent of hybridisation within Hungarian populations by including different marker types (SNPs) in the analysis and to take this into account when establishing the gene bank populations. Regardless of this, we found pure *C. carassius* (Kölked) or populations with only one or two hybrid individuals (Dunafalva, Cún-Szaporca) among the studied populations. Their further investigation would be important to identify factors that limit the spread of *C. gibelio*, thus preventing the development of hybridization introgression.

### Genetic diversity

Detailed data on the genetic diversity of populations of the species in Northern and Central Europe and England are already available^30,59,60^, but data on the Hungarian population is incomplete as only two samples from the area were included in Jeffries et al. (2016) study^57^. In the studies on crucian carp, partly with different microsatellites, the observed heterozygosity (^57^—range of 0.06–0.44, ^60^**—**Ho range of 0.03–0.40) was generally lower than the results obtained in the present study (range of 0.49–0.61). Considerably lower values were also observed for allelic richness (^30^—range of 1.21–2.95, ^60^—range of 1. 26–2.37) compared to our results (6.01–7.98). Jeffries et al. (2016) described a decreasing allelic richness tendency along an east-to-west longitudinal gradient in northern-central European populations^57^, a trend not observed in our study, since diversity values did not show significant differences between Hungarian populations. In our case, isolation by distance was also not detected between populations, probably due to the small geographical distances. However, this may change with larger numbers of (SNP) markers, as Jeffries et al. (2016) pointed out that RADseq data show a much stronger Isolation By Distance pattern than microsatellite markers^57^.

### Genetic structure

In our study, the pairwise F_ST_ values between populations ranged from 0.054 to 0.192, indicating low to medium differentiation. For the large-scale comparison between Northern and Central Europe, F_ST_ values averaged 0.43 (range of 0.0–0.864)^56^. When considering within-country values, within some country’s populations had low (Belgian 0.0–0.65) while in other cases they show a wide range (e.g., Sweden 0.084–0.838). The results of our study are most consistent with the Polish values (0.074–0.191).

However, the structure within the Hungarian population also showed segregation of the South-Danube-Drava region (Kölked, Dunafalva, Cún-Szaporca), between which the genetic separation was low and strong gene flow was detected. Interestingly, the Danube River had a significant isolation effect here, as the Baja population, geographically closest to them but located on the other side of the Danube River, showed the highest pairwise F_ST_ values with these populations, while the populations of Alag and Lake Kolon, located at a large distance, presented low F_ST_ values. Similarly, the population along the Mura Oxbow (which flows into the Drava River) showed a greater distance to these populations than to the geographically distant Lake Fertő. Although there are no other studies from the area for crucian carp, the genetic structure of the European mudminnow (*Umbra krameri*, Walbaum 1792) and tench (*Tinca tinca* Linnaeus 1758), which occur in the same habitats as the crucian carp, also shows the separating effect of the Danube River^61,62^. In the case of the European mudminnow, the populations east and west of the Danube River are separated and the eastern Lake Kolon showed a connection with the upper Danube (northern) populations. The European mudminnow populations along the Mura Oxbow River are connected with the geographically close Balaton populations north of them, but in contrast to the crucian carp, these populations are already separated from the northern Danube populations^61^. Similar to the crucian carp, the Cún-Szaporca population of tench showed much higher genetic divergence with Lake Kolon (F_ST_ = 0.138) than with the upper Danube population of Lake Fertő (F_ST_ = 0.054)^62^.

### Implications for conservation of the species

Among the areas we studied, the hybridization of Prussian carp in the South Danube areas was low. These populations contained few or no hybrid individuals. Examination of migration patterns revealed that these populations mostly served as source populations to surrounding habitats and showed high gene flow with each other. The low level of hybridisation is probably not only a result of isolation, but also of the quality of the habitats, and therefore the quality of the crucian carp population.

The diet of crucian carp is more associated with the bottom^63^ and primarily consists of primary consumers such as zoobenthos and zooplankton, which are shared resources for the two species in the presence of crucian carp^59^. The Prussian carp prefers to use lower trophic resources (plants, algal detritus) which the crucian carp does not^59^. Their co-occurrence leads to the Prussian carp outcompeting the native species, having better growth and reaching a larger body size in adults, which consequently leads to the extinction of the crucian carp^58^. Habitats with a rich littoral zone reduce niche overlap, and if more plant food is available, the Prussian carp feeding niche is shifted away from the native crucian carp^59^. It would be useful to evaluate the habitats studied from this perspective in the future to identify key factors that have ensured lower hybridization rates in the South Danube populations.

There was no evidence of a recent population bottleneck in any of the populations, but the Garza-Williamson M-ratio test resulted in low values in all of the natural populations indicating a recent reduction in the effective size of the populations. The slow reduction in effective population size may be a consequence of hybridization. Increased rates of hybridization of diploid *C. gibelio* males with *C. carassius* females in the area result in non-viable or sterile offspring, reducing the size of the next generation^23,63^. It would be necessary to investigate the sex ratio of *C. gibelio* populations in the habitats studied to get a more accurate picture of the expected future changes and to establish more effective management.

In Hungary, similar to the English^8^ and Czech^13^ experience, it has become necessary to initiate a crucian carp conservation programme. However, based on our results, this cannot be implemented in situ safely due to the high level of hybridisation, making it necessary to establish an *ex-situ* gene bank. The extreme (dry and warm) weather of recent years has often resulted in small water bodies drying out, making the populations of the species highly vulnerable. The high tolerance of hypoxic conditions in winter and high phenotypic plasticity in the presence of predatory species^64^, make the crucian carp suitable for colonizing small water bodies with low oxygen levels, even in the presence of predatory species that are tolerant of extreme conditions. Small shallow pit ponds^30^, whether new or previously cleared of Prussian carp and predators, with a sustainable water supply and sections of small channels can provide suitable habitat for reintroductions from gene bank stocks. The eight natural populations we studied, especially the South Danube populations, could provide a good basis for the establishment of a genetically controlled gene bank of remaining crucian carp stocks, without hybrids.

## Supplementary Information

Supplementary Information 1.

Supplementary Information 2.

## Data Availability

Sequence data that support the findings of this study have been deposited in the NCBI GenBank with the primary accession codes PQ584899—PQ585085. For further clarification contact the corresponding author.
