# Supplementary material for: Genetic survey of crucian carp Carassius carassius populations in Hungary for a conservation project to establish live gene bank
Source: Sci Rep. 2025 Mar 14;15:8835. doi: 10.1038/s41598-025-93468-6 (PMC11909271; doi:10.1038/s41598-025-93468-6)
Supplement: Supplementary file 2 — Supplementary Information 2. [file 41598_2025_93468_MOESM2_ESM.pdf]

Figure S1. The genetic structure of the nine crucian carp populations of Hungary based on the Neighbor-joining tree (A), Hierarchical STRUCTURE analysis first for K=3 (above dotted line) and for K=2 and K=3 (below dotted line) in the substructure (B), and DAPC analysis (C) performed on the microsatellite data. Populations: a - Alag, b - Baja, df - Dunafalva, f - Lake Fertő, c-sz - Cún-Szaporca, k – Kölked, kt - Lake Kolon, m - Mura Oxbow, rm- Rétimajor

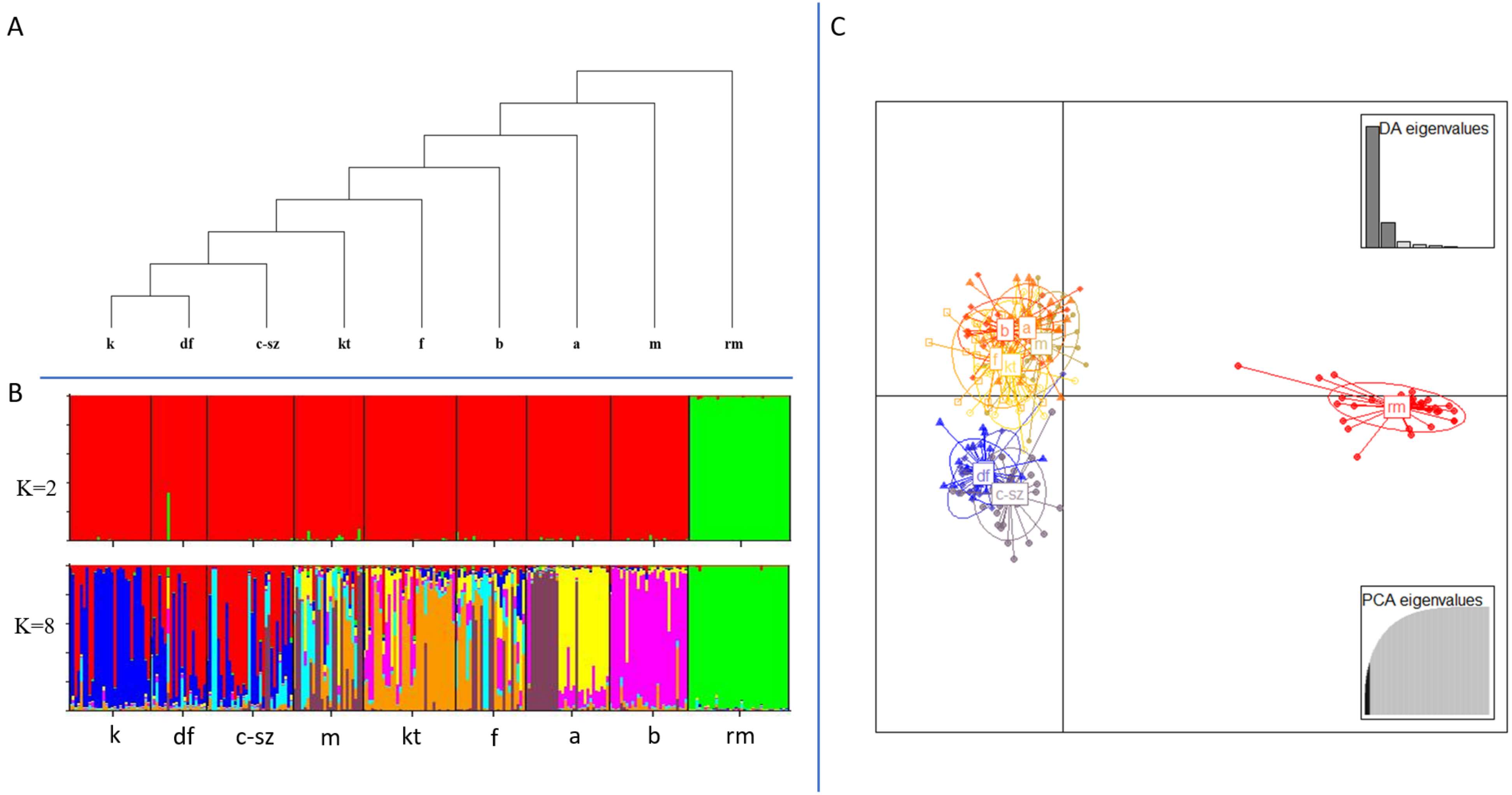

Figure S2. The most probable cluster number (K) based on the LnP(K) and Delta K values in the nine examined populations. The row highlighted in bold in the table represents the most probable cluster based on the ΔK method

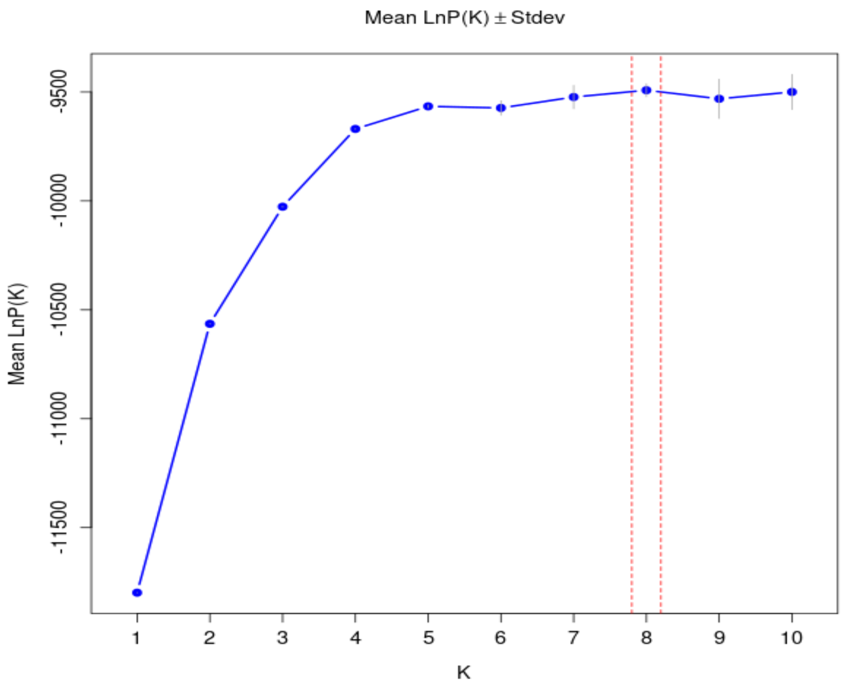

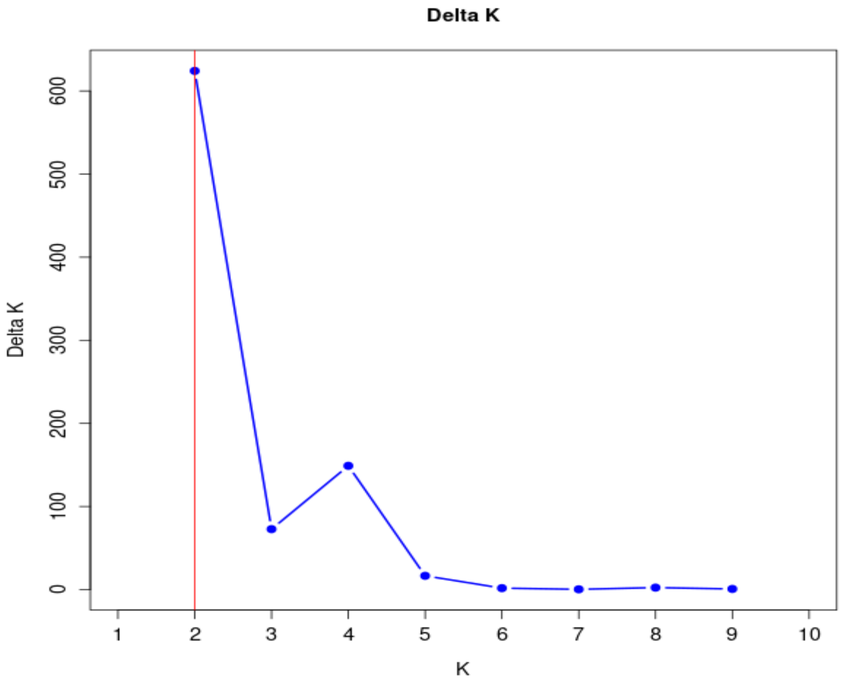

K	Reps	Mean LnP(K)	Stdev LnP(K)	Ln'(K)	|Ln''(K)|	Delta K
1	10	-11799.15000	0.60415	NA	NA	NA
**2**	**10**	**-10564.60000**	**1.11654**	**1234.55000**	**696.92000**	**624.17699**
3	10	-10026.97000	2.48061	537.63000	180.48000	72.75619
4	10	-9669.82000	1.70150	357.15000	253.44000	148.95070
5	10	-9566.11000	6.69136	103.71000	111.00000	16.58855
6	10	-9573.40000	32.77791	-7.29000	57.30000	1.74813
7	10	-9523.39000	53.49651	50.01000	18.75000	0.35049
8	10	-9492.13000	29.58115	31.26000	70.59000	2.38632
9	10	-9531.46000	89.99913	-39.33000	70.77000	0.78634
10	10	-9500.02000	80.47125	31.44000	NA	NA

Figure S3. The most probable cluster number (K) based on the LnP(K) and Delta K values in the eight natural populations . The row highlighted in bold in the table represents the most probable cluster based on the ΔK method

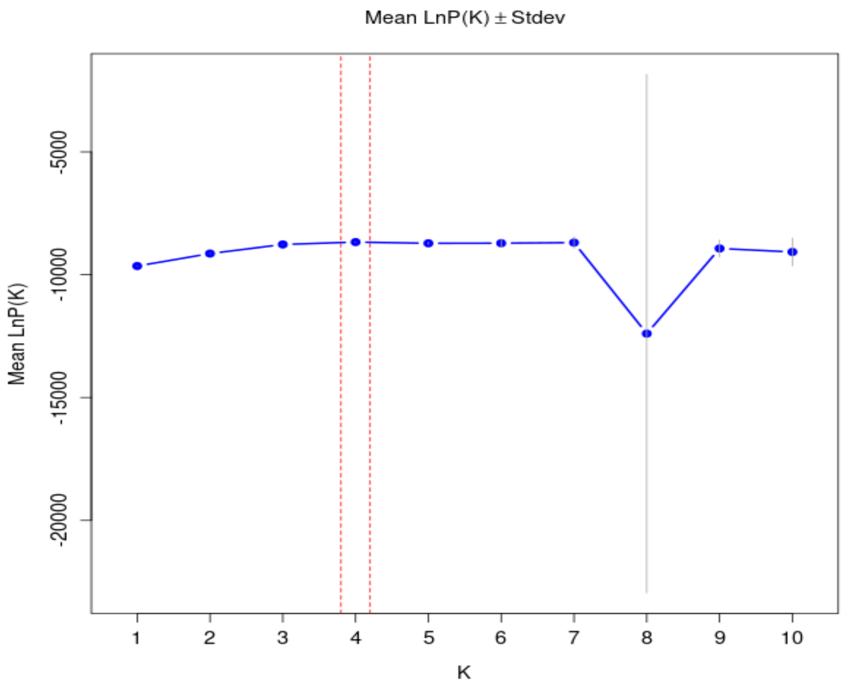

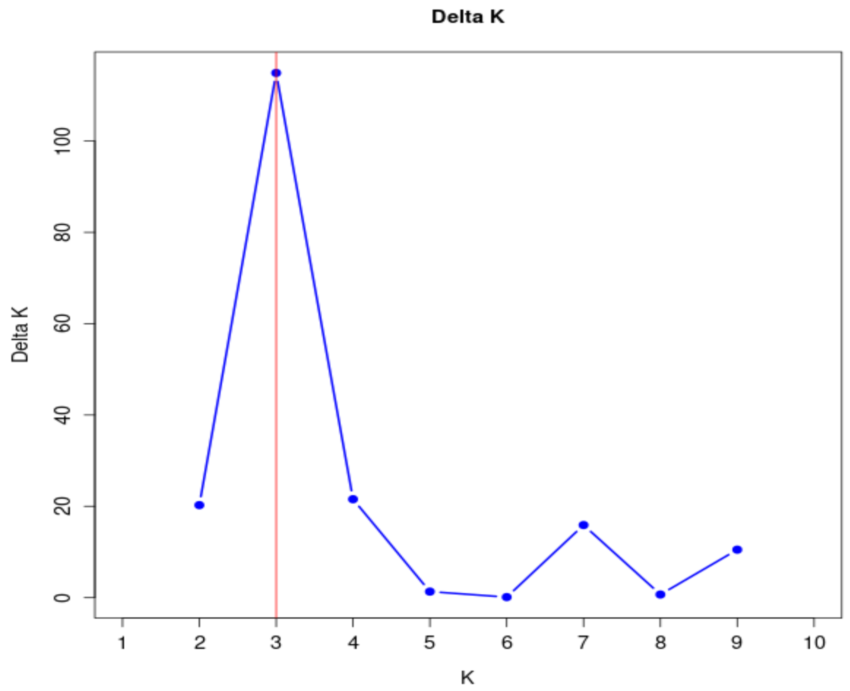

K	Reps	Mean LnP(K)	Stdev LnP(K)	Ln'(K)	|Ln''(K)|	Delta K
1	10	-9646.79000	0.64885	NA	NA	NA
2	10	-9138.99000	6.78306	507.80000	137.40000	20.25635
**3**	**10**	**-8768.59000**	**2.38442**	**370.40000**	**274.02000**	**114.92117**
4	10	-8672.21000	6.76403	96.38000	145.77000	21.55076
5	10	-8721.60000	40.34543	-49.39000	53.15000	1.31737
6	10	-8717.84000	179.10235	3.76000	20.34000	0.11357
7	10	-8693.74000	234.60260	24.10000	3728.44000	15.89258
8	10	-12398.08000	10550.30597	-3704.34000	7169.60000	0.67956
9	10	-8932.82000	343.43836	3465.26000	3606.59000	10.50142
10	10	-9074.15000	563.29895	-141.33000	NA	NA

Figure S4. The most probable cluster number (K) based on the LnP(K) and Delta K values in populations k, df and c-sz . The row highlighted in bold in the table represents the most probable cluster based on the ΔK method

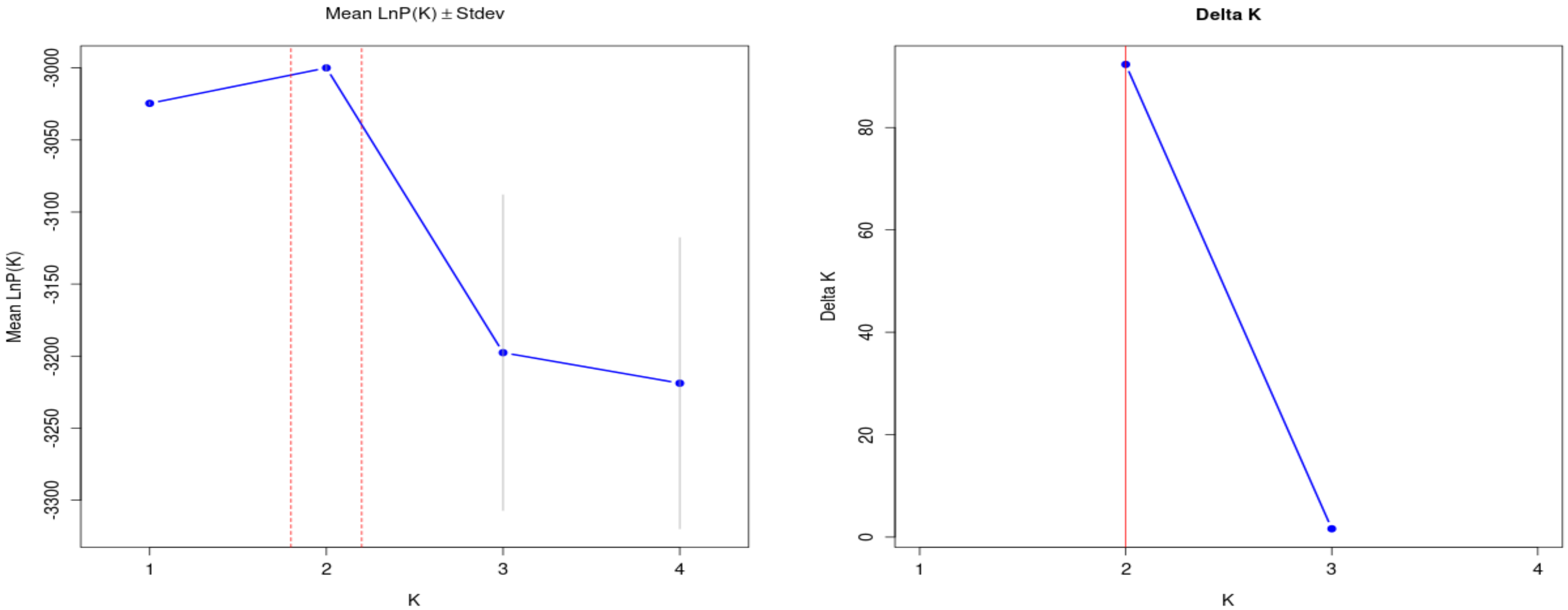

K	Reps	Mean LnP(K)	Stdev LnP(K)	Ln'(K)	|Ln''(K)|	Delta K
1	10	-3024.60000	1.05830	NA	NA	NA
**2**	**10**	**-2999.99000**	**2.40668**	**24.61000**	**222.22000**	**92.33465**
3	10	-3197.60000	109.51074	-197.61000	176.39000	1.61071
4	10	-3218.82000	100.99156	-21.22000	NA	NA

Figure S5. The most probable cluster number (K) based on the LnP(K) and Delta K values in populations m, kt, f, a, and b . The row highlighted in bold in the table represents the most probable cluster based on the ΔK method

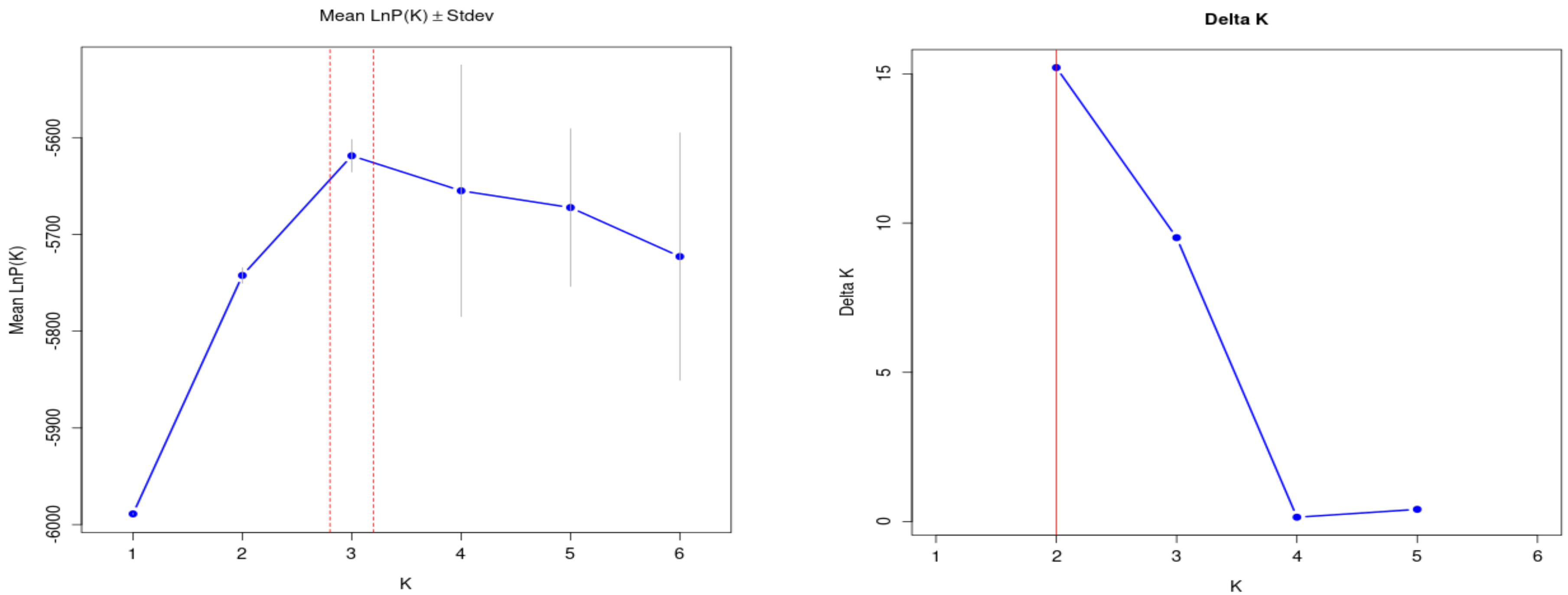

K	Reps	Mean LnP(K)	Stdev LnP(K)	Ln'(K)	|Ln''(K)|	Delta K
1	10	-5988.82000	0.75100	NA	NA	NA
**2**	**10**	**-5742.42000**	**8.06898**	**246.40000**	**122.73000**	**15.21010**
3	10	-5618.75000	16.80220	123.67000	159.82000	9.51185
4	10	-5654.90000	129.96529	-36.15000	18.79000	0.14458
5	10	-5672.26000	81.46743	-17.36000	33.27000	0.40838
6	10	-5722.89000	127.87009	-50.63000	NA	NA

Table S1. Sampling locations and sample sizes.

Location	Label	GPS coordinate		Sample size
**Lake Kolon**	kt	N 46°45'39"	E 19°20'28"	33
**Rétimajor**	rm	N 46°48'26"	E 18°35'24"	36
**Baja**	b	N 46°11'40"	E 18°56'07"	28
**Lake Fertő**	f	N 47°37'24"	E 16°45'23"	25
**Alag**	a	N 47°38'57"	E 19°10'08"	30
**Kölked**	k	N 45°56'16"	E 18°43'32"	29
**Mura River**	m	N 46°22'52"	E 16°46'39"	25
**Dunafalva**	df	N 46°06'28"	E 18°47'44"	20
**Cun-Szaporca**	c-sz	N 45°48'00"	E 18°09'06"	31

Table S2. Demographic data of the based on COI sequences in the nine Hungarian crucian carp populations.

Stock	Fu's F_S	Tajima's D	Obs Nh	Chakraborty's test	
				Exp Nh	P
**Lake Kolon**	-2.176	-1.17776	8	4.6	0.049*
**Rétimajor**	-2.257	-1.28103	11	6.4	0.021*
**Baja**	-1.441	-0.88999	7	6.2	0.435
**Lake Fertő**	2.975	2.20685	8	6.3	0.214
**Alag**	2.718	0.99119	11	7.9	0.098
**Kölked**	1.311	1.26176	2	2.9	0.883
**Mura river**	6.301	2.36574	10	9.2	0.429
**Dunafalva**	3.620	-2.08915***	5	4.6	0.535
**Cun-Szaporca**	3.367	-0.87395	9	4.8	0.017*

ObsNh— Observed number of haplotypes, Exp Nh—Expected number of haplotypes, P—P (k or more alleles), Statistical significance: * P<0.05; ***<0.001.

Table S3. The Garza-Williamson ratio in the natural populations

Locus#	Alag	Baja	Dunafalva	Lake Fertő	Cun-Szaporca	Kölked	Lake Kolon	Mura River	Mean	s.d.
1	0.60000	1.00000	0.66667	0.66667	0.66667	1.00000	0.66667	0.60000	0.73333	0.16714
2	0.57143	0.60000	0.66667	0.66667	0.60000	0.60000	0.60000	0.60000	0.61310	0.03450
3	0.29787	0.34146	0.23529	0.26230	0.27027	0.29412	0.18868	0.25532	0.26816	0.04556
4	0.36364	0.23077	0.23077	0.36364	0.13043	0.18182	0.23077	0.27273	0.25057	0.08141
5	0.13333	0.13333	0.13793	0.20000	0.23810	0.33333	0.13333	0.13333	0.18034	0.07345
6	0.53846	0.57143	0.60000	0.45455	0.60000	0.66667	0.55556	0.45455	0.55515	0.07294
7	0.15942	0.18367	0.26829	0.16438	0.24528	0.20000	0.13580	0.16471	0.19020	0.04550
8	0.38462	0.28571	0.26087	0.26667	0.38095	0.30435	0.30435	0.30769	0.31190	0.04709
9	0.08911	0.33333	0.33333	0.07080	0.06838	0.30769	0.04938	0.12329	0.17191	0.12858
10	0.14815	0.17978	0.18557	0.16471	0.22581	0.19588	0.20253	0.18557	0.18600	0.02355
11	0.11200	0.21176	0.23077	0.16495	0.24706	0.21918	0.19481	0.16832	0.19361	0.04366
12	0.08584	0.09434	0.08059	0.13757	0.12648	0.11892	0.09665	0.10035	0.10509	0.02030
13	0.15789	0.40000	1.00000	0.22727	0.21053	1.00000	0.28571	0.21053	0.43649	0.35510
Mean	0.28014	0.29713	0.32473	0.29309	0.30846	0.31109	0.28033	0.27511	0.29626	0.01753
s.d.	0.19216	0.16160	0.20371	0.19319	0.19506	0.17276	0.20033	0.17181	0.18633	0.01541

Table S4. Hybrid individuals indicated based on the fragment size of Gf1 and Gf29 microsatellites. (C. gibelio fargment size is marked with bold, * indicates hybrid based on the COI sequence)

sample	pop	Gf1	Gf1	Gf29	Gf29	mtDNA
282	a	**300**	**302**	**203**	217	
283	a	298	**300**	**191**	219	
284	a	298	**300**	**203**	225	
285	a	**300**	**302**	**193**	213	*
286	a	**300**	**302**	**203**	225	
287	a	**302**	**302**	**203**	**203**	
288	a	**300**	**302**	**203**	237	
289	a	**300**	**300**	**199**	**203**	
290	a	**300**	**302**	**203**	235	
291	a	0	0	0	0	
292	a	**300**	**302**	**203**	221	*
293	a	**300**	**302**	**203**	225	*
294	a	**300**	**300**	219	237	
295	a	**300**	**300**	225	237	*
296	a	**300**	**300**	219	221	*
297	a	**300**	**300**	221	221	
298	a	**300**	**300**	221	237	
299	a	**300**	**300**	225	237	
300	a	**300**	**302**	221	221	
301	a	**300**	**300**	215	221	
302	a	**300**	**300**	219	221	
303	a	**300**	**300**	221	237	
304	a	**300**	**300**	221	225	
305	a	**300**	**300**	215	231	
306	a	**300**	**300**	219	225	
307	a	**300**	**300**	225	237	
308	a	**300**	**300**	225	237	
309	a	**300**	**300**	**201**	221	
310	a	**300**	**300**	221	225	
311	a	0	0	0	0	
254	b	0	0	227	227	
255	b	**300**	**300**	227	227	
256	b	**300**	**300**	219	227	
257	b	**300**	**300**	227	227	
258	b	**300**	**300**	**199**	223	
259	b	**300**	**300**	**203**	227	
260	b	**300**	**300**	**197**	221	
261	b	**300**	**300**	**207**	219	
262	b	**300**	**300**	**197**	221	
263	b	**300**	**300**	**197**	221	
264	b	**300**	**300**	**197**	221	
265	b	**300**	**300**	0	0	
266	b	**300**	**300**	219	227	
267	b	**300**	**300**	189	219	
268	b	**300**	**300**	209	221	
269	b	**300**	**300**	**197**	221	

270	b	**300**	**300**	209	227	
271	b	**300**	**300**	**191**	219	
272	b	**300**	**300**	**207**	227	
273	b	**300**	**300**	**187**	219	
274	b	**300**	**300**	**197**	221	
275	b	**300**	**300**	**197**	221	
276	b	**300**	**300**	**189**	227	
277	b	**300**	**300**	**189**	227	
278	b	**300**	**300**	**205**	219	
279	b	**300**	**300**	**189**	219	
280	b	**300**	**300**	**189**	219	
281	b	**300**	**300**	219	225	
32	df	298	298	213	213	
33	df	298	298	215	215	
34	df	298	298	219	219	
35	df	298	298	213	213	
36	df	298	298	213	213	
37	df	298	298	213	215	
38	df	**300**	**300**	0	0	
39	df	298	298	213	215	
40	df	298	298	215	229	
41	df	298	298	213	215	
42	df	298	298	213	213	
43	df	298	298	213	219	
44	df	298	298	215	229	
45	df	298	298	213	215	
46	df	298	298	213	215	*
47	df	298	298	213	215	
48	df	298	298	213	215	
49	df	298	298	213	215	
50	df	298	298	215	229	
51	df	298	298	215	215	
130	f	0	0	0	0	
131	f	**300**	**300**	219	219	
132	f	**300**	**300**	221	225	
133	f	**300**	**300**	217	217	
134	f	**300**	**300**	225	233	
135	f	**300**	**300**	221	221	
136	f	0	0	**199**	223	
137	f	298	298	**203**	217	
138	f	298	298	217	235	
139	f	298	298	217	225	*
140	f	**300**	**300**	213	219	
141	f	298	**300**	**197**	231	*
142	f	298	**300**	**203**	219	*
143	f	298	**300**	**203**	217	
144	f	298	298	217	225	
145	f	**300**	**300**	215	221	
146	f	298	298	219	219	
147	f	298	**300**	**193**	225	*

148	f	298	298	217	233	
149	f	0	0	253	253	
150	f	0	0	**203**	219	*
151	f	**300**	**300**	219	235	
152	f	0	0	0	0	
153	f	**300**	**300**	229	229	
154	f	**300**	**300**	221	221	
1	csz	298	298	215	215	
2	csz	298	298	229	229	
3	csz	298	**300**	**197**	225	
4	csz	298	300	**197**	225	
5	csz	298	298	213	215	
6	csz	298	298	215	229	*
7	csz	298	298	213	215	
8	csz	298	298	213	215	
9	csz	298	298	219	229	
10	csz	298	298	215	219	
11	csz	298	298	215	229	
12	csz	298	298	213	229	
13	csz	298	298	215	215	
14	csz	298	298	215	215	
15	csz	298	298	215	215	
16	csz	298	**300**	**199**	229	
17	csz	298	298	215	229	
18	csz	298	298	213	229	*
19	csz	298	298	215	229	*
20	csz	298	298	213	219	
21	csz	298	298	215	215	
22	csz	298	**300**	205	229	
23	csz	298	**300**	207	213	
24	csz	298	298	219	229	
25	csz	298	298	215	229	
26	csz	298	298	213	229	
27	csz	0	0	0	0	
28	csz	298	298	229	233	
29	csz	298	298	225	229	
30	csz	298	298	215	229	
31	csz	298	298	219	229	
52	k	298	298	213	229	
53	k	298	298	213	219	
54	k	298	298	213	229	
55	k	298	298	213	215	
56	k	298	298	219	229	
57	k	298	298	215	215	
58	k	298	298	213	213	
59	k	298	298	213	213	
60	k	298	298	213	215	
61	k	298	298	215	215	
62	k	298	298	213	219	
63	k	298	298	213	215	

64	k	298	298	215	221
65	k	298	298	219	229
66	k	298	298	215	229
67	k	298	298	219	229
68	k	298	298	213	215
69	k	298	298	215	219
70	k	298	298	219	229
71	k	298	298	213	229
72	k	298	298	213	221
73	k	298	298	213	215
74	k	298	298	219	229
75	k	298	298	219	219
76	k	298	298	215	229
77	k	298	298	213	213
78	k	298	298	213	215
79	k	298	298	215	219
80	k	298	298	215	219
155	kt	0	0	0	0
156	kt	**300**	**300**	221	237
157	kt	**300**	**300**	215	215
158	kt	**300**	**300**	225	237
159	kt	**300**	**300**	215	217
160	kt	**300**	**300**	219	219
161	kt	**300**	**300**	217	217
162	kt	**300**	**300**	215	215
163	kt	**300**	**300**	219	219
164	kt	**300**	**300**	219	219
165	kt	0	0	253	253
166	kt	**300**	**300**	219	219
167	kt	**300**	**300**	237	237
168	kt	**300**	**300**	219	221
169	kt	0	0	215	217
170	kt	298	298	217	221
171	kt	0	0	0	0
172	kt	**300**	**300**	219	235
173	kt	298	298	215	231
174	kt	**300**	**300**	217	219
175	kt	298	298	217	235
176	kt	298	298	217	219
177	kt	0	0	**201**	215
178	kt	298	298	217	221
179	kt	298	298	217	217
180	kt	298	298	217	235
181	kt	298	298	215	231
182	kt	298	298	**201**	219
183	kt	298	298	217	217
184	kt	298	298	221	225
185	kt	298	298	215	217
186	kt	298	298	**201**	221
187	kt	298	298	217	219

312	m	**300**	**300**	235	239	
313	m	**300**	**300**	**205**	221	*
314	m	**300**	**302**	**203**	225	
315	m	**300**	**300**	221	237	
316	m	**300**	**300**	225	237	
317	m	**300**	**300**	231	231	
318	m	**300**	**300**	221	237	*
319	m	0	0	0	0	*
320	m	**300**	**300**	199	239	
321	m	**300**	**302**	203	239	*
322	m	298	298	217	235	*
323	m	298	298	0	0	
324	m	**300**	**300**	217	235	
325	m	**300**	**300**	221	221	
326	m	298	298	203	225	
327	m	298	**300**	205	235	
328	m	**300**	**300**	219	221	*
329	m	298	300	193	219	*
330	m	298	298	217	235	*
331	m	0	0	0	0	
332	m	298	298	217	225	
333	m	298	298	225	235	
334	m	298	**300**	219	219	
335	m	298	**300**	**205**	219	
336	m	**300**	**300**	**203**	**205**	*

Table S5. Pairwise Fst with the ENA correction (below the diagonal) and the Cavalli-Sforza and Edwards genetic distances with the INA correction (above the diagonal).

population	Alag	Baja	Dunafalva	Lake Fertő	Cún-Szaporca	Kölked	Lake Kolon	Mura River
Alag		0.391	0.510	0.419	0.504	0.520	0.400	0.406
Baja	0.055		0.417	0.414	0.469	0.423	0.321	0.449
Dunafalva	0.156	0.163		0.421	0.318	0.285	0.360	0.492
Lake Fertő	0.055	0.054	0.089		0.444	0.439	0.367	0.361
Cún-Szaporca	0.173	0.192	0.039	0.105		0.317	0.422	0.451
Kölked	0.189	0.189	0.014	0.108	0.059		0.390	0.512
Lake Kolon	0.063	0.069	0.082	0.038	0.105	0.099		0.397
Mura	0.068	0.113	0.165	0.051	0.131	0.190	0.070	

Table S6. Relative directional Migration matrix calculated using D (Jost, 2008) statistically significant values (alpha = 0.05) are indicated with '*'

Populations	Alag	Baja	Dunafalva	Lake Fertő	Cún-Szaporca	Kölked	Lake Kolon	Mura River
Alag	NA	0.287	0.116	0.195	0.158	0.111	0.316	0.383
Baja	0.463	NA	0.363	0.315	0.276*	0.212	0.634	0.291
Dunafalva	0.342*	0.335	NA	0.454	0.979	0.901	0.454*	0.512*
Lake Fertő	0.426*	0.344	0.246	NA	0.234	0.215	0.55	0.558
Cún-Szaporca	0.147	0.149	0.601	0.243	NA	0.488	0.268	0.445*
Kölked	0.372*	0.253	0.910	0.581*	1.000	NA	0.478*	0.530*
Lake Kolon	0.322	0.639	0.244	0.388	0.192	0.296	NA	0.384
Mura River	0.308	0.197	0.108	0.378	0.225	0.098	0.256	NA

Table S7. The raw microsatellite data of the eight natural Carassius populations

Sample ID	pop	Gf1	Gf1	YJ10	YJ10	Gf29	Gf29	YJ0022	YJ0022	MFW7	MFW7	CypG24	CypG24	HLJYJ017	HLJYJ017	J62	J62	HLJYJ029	HLJYJ029	HLJYJ028	HLJYJ028	HLJYJ046	HLJYJ046	HLJYJ041	HLJYJ041	HLJYJ082	HLJYJ082
282	a	317	319	167	167	220	234	176	182	164	178	176	186	232	272	0	0	113	165	403	403	179	239	262	370	0	0
283	a	315	317	169	169	208	236	176	182	164	178	178	178	288	288	0	0	121	213	371	371	223	263	286	314	0	0
284	a	315	317	167	167	220	242	176	182	164	178	182	184	264	280	0	0	125	189	383	383	223	247	222	358	0	0
285	a	317	319	169	169	210	230	176	186	164	178	176	186	260	288	0	0	125	189	383	383	247	299	286	414	192	198
286	a	317	319	167	167	220	242	176	176	164	178	176	178	232	296	0	0	121	181	383	383	227	299	314	362	0	0
287	a	319	319	167	167	220	220	176	184	164	164	0	0	280	288	0	0	0	0	0	0	187	187	0	0	0	0
288	a	317	319	169	169	220	254	176	182	164	178	176	186	0	0	0	0	121	181	383	383	227	263	226	362	0	0
289	a	317	317	169	169	216	220	186	186	164	164	176	178	232	264	0	0	173	189	355	383	231	303	222	246	198	198
290	a	317	319	167	171	220	252	176	186	164	164	176	186	232	296	0	0	125	181	383	383	227	263	222	418	192	198
291	a	0	0	0	0	0	0	0	0	0	0	178	182	264	300	0	0	125	189	367	367	299	299	286	362	198	198
292	a	317	319	169	173	220	238	176	176	164	164	174	186	264	296	0	0	121	189	367	367	299	299	286	418	180	198
293	a	317	319	169	171	220	242	176	176	164	178	174	186	264	284	0	0	113	181	355	355	247	247	226	354	180	198
294	a	317	317	169	171	236	254	176	176	164	178	186	186	284	292	189	193	125	125	355	391	227	259	334	358	198	198
295	a	317	317	169	171	242	254	176	176	164	164	178	186	272	284	199	199	125	125	355	391	239	255	350	418	198	198
296	a	317	317	167	171	236	238	176	176	164	164	178	178	284	292	193	193	125	125	379	411	243	247	338	350	198	198
297	a	317	317	169	171	238	238	176	176	164	178	178	178	272	288	193	199	125	125	0	0	0	0	0	0	0	0
298	a	317	317	167	169	238	254	176	176	164	178	184	186	288	288	193	193	121	125	375	375	227	239	350	362	198	198
299	a	317	317	167	169	242	254	176	176	164	178	184	186	284	296	193	199	125	125	375	383	259	259	354	358	198	198
300	a	317	319	169	171	238	238	176	176	164	178	176	178	236	300	0	0	121	169	379	379	227	227	414	414	198	198
301	a	317	317	169	171	232	238	176	176	164	178	178	186	272	284	193	193	125	125	355	419	223	243	406	418	198	198
302	a	317	317	169	171	236	238	176	176	164	178	178	184	284	300	195	195	121	125	0	0	227	243	418	454	198	198
303	a	317	317	167	171	238	254	176	176	164	164	0	0	0	0	0	0	0	0	391	391	243	243	382	410	198	198
304	a	317	317	167	171	238	242	176	186	164	178	178	178	288	296	195	195	121	125	0	0	215	299	338	418	198	198
305	a	317	317	167	171	232	248	176	176	164	164	178	184	280	292	187	193	125	125	339	391	247	255	302	338	198	198
306	a	317	317	169	171	236	242	176	176	164	178	178	184	288	300	199	199	121	125	371	375	247	259	286	338	198	198
307	a	317	317	167	171	242	254	176	176	164	178	178	186	284	300	193	193	125	125	391	407	239	247	334	406	198	198
308	a	317	317	169	171	242	254	176	186	164	178	178	186	272	288	193	193	125	125	339	375	243	299	302	350	198	198
309	a	317	317	169	171	218	238	176	176	164	178	178	184	280	288	187	193	121	121	355	379	215	259	406	406	198	198
310	a	317	317	169	171	238	242	176	176	164	178	178	178	272	288	193	199	125	125	339	375	239	243	362	414	198	198
311	a	0	0	0	0	0	0	0	0	0	0	178	180	272	292	187	193	125	125	391	407	227	259	382	382	198	198
254	b	0	0	167	169	244	244	176	176	164	178	178	178	280	288	199	199	125	129	367	407	203	219	326	362	198	198
255	b	317	317	169	169	244	244	176	176	164	178	178	184	288	300	193	199	125	125	371	411	195	271	390	478	198	198
256	b	317	317	169	169	236	244	176	176	164	164	178	180	272	272	199	199	125	129	323	323	263	263	354	398	194	198
257	b	317	317	169	171	244	244	176	186	164	178	178	178	272	272	187	193	125	125	399	407	211	211	334	354	198	198
258	b	317	317	167	169	216	240	176	176	164	178	178	178	272	288	187	195	125	125	371	407	203	227	478	514	198	198
259	b	317	317	169	169	220	244	174	176	164	178	178	178	284	288	193	199	125	125	367	387	267	275	318	542	198	198
260	b	317	317	167	169	214	238	176	176	164	178	178	178	284	292	189	195	0	0	359	367	243	247	362	418	198	198
261	b	317	317	167	169	224	236	176	176	164	178	178	178	272	284	193	199	129	129	371	407	231	275	478	490	198	198
262	b	317	317	167	167	214	238	176	176	164	178	178	178	276	288	193	199	125	125	371	375	251	267	318	370	198	198
263	b	317	317	169	169	214	238	176	186	164	178	178	178	272	288	199	199	121	125	355	367	251	263	318	358	198	198
264	b	317	317	169	169	214	238	176	176	164	178	178	180	280	280	199	199	125	125	371	391	239	267	318	370	198	198
265	b	317	317	169	171	0	0	176	176	164	164	178	180	272	300	195	199	121	129	359	399	251	267	386	390	198	198
266	b	317	317	169	169	236	244	176	176	164	178	178	184	280	284	199	207	121	125	323	367	235	259	306	398	198	198
267	b	317	317	169	171	206	236	176	176	164	178	178	178	276	296	187	199	125	125	371	387	215	259	354	366	198	198
268	b	317	317	169	169	226	238	176	176	164	178	178	178	272	284	189	195	125	129	371	387	271	275	350	354	198	198
269	b	317	317	169	171	214	238	176	176	164	178	178	178	272	320	193	193	125	129	379	411	219	239	490	490	198	198
270	b	317	317	169	171	226	244	176	176	164	178	178	178	276	288	193	193	125	125	351	371	271	275	334	498	198	198
271	b	317	317	169	169	208	236	176	176	164	178	178	178	288	292	193	199	125	125	359	375	235	267	350	490	198	198
272	b	317	317	169	171	224	244	176	176	164	178	178	178	272	288	193	193	125	125	371	375	243	263	358	390	198	198
273	b	317	317	169	169	204	236	176	186	164	164	178	182	284	296	187	193	125	129	371	379	267	271	354	390	194	198

274	b	317	317	169	169	214	238	176	176	164	178	178	178	284	300	193	193	125	125	363	371	211	259	322	362	198	198
275	b	317	317	169	169	214	238	176	176	164	178	178	178	280	296	193	199	121	125	387	395	259	259	354	406	198	198
276	b	317	317	167	169	206	244	176	176	164	178	178	178	280	284	187	193	121	125	351	367	247	275	334	354	198	198
277	b	317	317	171	171	206	244	176	176	164	178	178	178	288	288	193	193	121	129	375	399	247	251	422	566	198	198
278	b	317	317	169	171	222	236	176	186	164	178	178	178	280	296	193	199	125	125	371	407	227	267	418	478	198	198
279	b	317	317	167	169	206	236	176	176	164	178	178	178	272	292	193	193	125	125	355	367	211	211	314	390	198	198
280	b	317	317	167	169	206	236	176	176	164	178	178	178	272	288	193	193	121	125	371	379	211	243	302	390	194	198
281	b	317	317	169	169	236	242	176	186	164	178	178	178	276	288	199	199	125	125	335	367	275	279	322	478	198	198
32	df	315	315	167	167	230	230	176	176	178	178	178	182	292	308	193	199	125	125	351	407	211	239	422	506	198	198
33	df	315	315	167	167	232	232	176	186	0	0	178	180	272	272	193	199	125	125	363	407	239	243	334	370	198	198
34	df	315	315	167	169	236	236	176	176	178	178	178	178	276	288	187	207	121	125	327	415	211	211	474	534	198	198
35	df	315	315	167	169	230	230	176	186	178	184	178	178	284	296	199	199	125	129	359	379	235	259	358	482	198	198
36	df	315	315	167	169	230	230	176	176	178	178	178	178	288	292	193	199	125	129	331	415	223	235	342	518	198	198
37	df	315	315	169	169	230	232	176	176	178	178	178	180	288	292	193	199	125	129	375	391	219	223	354	482	198	198
38	df	317	317	0	0	0	0	0	0	162	162	0	0	0	0	0	0	0	0	0	0	0	0	0	0	0	0
39	df	315	315	167	169	230	232	176	176	178	178	178	178	284	296	187	209	125	125	355	367	211	271	334	470	198	198
40	df	315	315	169	169	232	246	176	186	178	178	178	178	272	280	199	199	125	125	335	407	215	259	358	362	198	198
41	df	315	315	167	167	230	232	176	176	178	184	178	180	280	292	193	199	121	121	0	0	255	263	350	354	198	198
42	df	315	315	167	167	230	230	176	176	178	178	178	178	280	296	193	199	125	129	339	343	207	239	358	414	198	198
43	df	315	315	167	167	230	236	176	186	178	178	178	178	268	296	193	193	125	125	371	391	211	211	362	422	198	198
44	df	315	315	169	169	232	246	176	186	178	190	178	178	280	300	199	199	125	125	331	355	255	259	338	370	198	198
45	df	315	315	167	169	230	232	176	186	178	178	178	182	276	288	187	193	125	125	367	391	251	255	326	326	198	198
46	df	315	315	169	169	230	232	176	186	0	0	178	180	276	300	193	193	129	129	0	0	219	227	442	574	198	198
47	df	315	315	167	169	230	232	176	176	178	178	178	178	288	304	193	199	125	125	355	363	235	251	346	358	198	198
48	df	315	315	167	167	230	232	174	176	178	178	178	178	268	304	193	199	125	129	327	363	259	263	334	354	198	198
49	df	315	315	167	169	230	232	176	176	178	178	178	178	280	292	187	207	125	125	355	387	251	263	318	470	198	198
50	df	315	315	167	169	232	246	176	176	178	178	178	178	284	300	191	193	125	125	363	391	235	243	326	354	198	198
51	df	315	315	167	169	232	232	176	176	178	178	178	178	292	292	193	199	125	125	371	423	231	231	302	302	198	198
130	f	0	0	0	0	0	0	0	0	0	0	178	178	300	300	199	199	125	125	0	0	271	287	0	0	198	198
131	f	317	317	167	167	236	236	176	186	164	178	178	178	284	288	199	199	125	125	335	343	207	255	350	354	198	198
132	f	317	317	167	169	238	242	176	176	164	178	178	178	284	308	199	199	125	125	355	359	207	227	310	402	198	201
133	f	317	317	169	169	234	234	176	186	164	178	178	178	284	288	193	193	125	125	375	399	267	283	346	366	198	198
134	f	317	317	169	169	242	250	176	186	0	0	178	178	292	296	199	199	125	125	327	411	211	255	0	0	198	198
135	f	317	317	167	169	238	238	176	176	164	178	178	178	276	292	207	207	113	125	355	399	207	207	302	354	198	198
136	f	0	0	0	0	216	240	0	0	164	164	172	178	236	288	0	0	125	181	331	399	0	0	274	426	0	0
137	f	315	315	167	169	220	234	176	176	164	164	176	178	284	288	0	0	125	181	375	375	227	227	258	326	0	0
138	f	315	315	167	167	234	252	176	176	164	178	178	178	288	292	193	199	125	125	331	355	235	235	294	418	198	198
139	f	315	315	167	169	234	242	176	176	164	178	176	178	260	280	0	0	125	173	395	395	303	303	262	358	0	0
140	f	317	317	167	167	230	236	176	176	164	176	178	182	276	280	0	0	125	201	375	375	295	295	254	422	192	198
141	f	315	317	167	167	214	248	176	178	164	178	178	178	268	276	0	0	125	161	363	363	0	0	254	338	192	198
142	f	315	317	167	167	220	236	176	176	164	178	178	178	280	288	0	0	125	225	355	355	227	227	318	442	180	198
143	f	315	317	169	169	220	234	176	176	164	178	176	178	236	300	0	0	125	161	383	383	227	227	318	406	180	198
144	f	315	315	167	169	234	242	176	176	164	178	178	180	284	304	199	199	125	125	359	359	207	255	382	438	198	198
145	f	317	317	169	169	232	238	176	176	164	178	178	178	280	280	199	199	125	125	0	0	0	0	0	0	0	0
146	f	315	315	169	169	236	236	176	186	164	178	178	180	280	288	193	195	125	125	363	375	239	239	390	406	198	198
147	f	315	317	169	169	210	242	176	178	164	178	172	178	0	0	0	0	125	189	331	335	235	259	318	334	192	198
148	f	315	315	167	169	234	250	176	186	164	178	178	178	284	288	199	199	125	125	359	399	243	271	302	390	198	198
149	f	0	0	0	0	270	270	176	184	164	178	178	178	288	292	199	199	125	125	351	375	239	267	338	406	198	198
150	f	0	0	0	0	220	236	186	186	164	164	178	178	236	280	0	0	125	161	355	355	227	255	270	418	186	198
151	f	317	317	169	169	236	252	176	186	164	164	178	178	284	296	193	207	125	125	327	331	211	255	294	338	198	198
152	f	0	0	0	0	0	0	0	0	0	0	0	0	0	0	193	199	125	125	0	0	0	0	0	0	0	0
153	f	317	317	169	169	246	246	176	176	164	178	178	178	284	284	199	199	125	125	331	399	215	259	406	406	198	198
154	f	317	317	167	169	238	238	176	176	164	178	178	180	280	308	193	199	125	125	331	387	231	243	294	310	198	198
1	c-sz	315	315	167	169	232	232	176	186	178	184	178	178	272	292	193	199	125	125	355	383	235	239	334	470	198	198
2	c-sz	315	315	169	169	246	246	186	186	178	178	178	178	288	300	199	199	125	125	335	335	227	239	334	450	198	198

3	c-sz	315	317	167	167	214	242	186	186	184	184	178	180	260	280	0	0	125	233	351	351	219	219	258	470	198	198
4	c-sz	315	317	167	167	214	242	186	186	184	184	178	180	260	280	0	0	125	233	351	351	219	219	258	470	198	198
5	c-sz	315	315	167	167	230	232	176	176	178	178	178	178	272	280	191	193	125	125	383	387	223	227	330	342	198	198
6	c-sz	315	315	167	167	232	246	186	186	178	178	178	178	280	292	199	207	125	129	379	399	219	227	350	462	198	198
7	c-sz	315	315	169	169	230	232	176	176	178	178	178	178	280	300	191	199	121	125	339	379	215	271	326	470	198	198
8	c-sz	315	315	167	167	230	232	186	186	178	178	178	178	276	280	191	195	125	125	343	379	203	215	410	482	198	198
9	c-sz	315	315	167	169	236	246	186	186	178	178	178	178	280	280	193	199	125	125	371	379	223	231	342	342	198	198
10	c-sz	315	315	169	171	232	236	176	186	178	178	178	178	276	300	193	199	125	125	363	371	215	275	458	486	198	198
11	c-sz	315	315	169	169	232	246	186	186	178	184	178	178	284	304	199	199	125	125	391	411	251	271	310	334	198	198
12	c-sz	315	315	167	167	230	246	176	186	178	178	178	178	276	296	199	199	125	125	379	387	211	263	442	470	198	198
13	c-sz	315	315	167	167	232	232	176	186	178	184	178	178	280	292	187	191	125	125	371	407	215	231	342	354	198	198
14	c-sz	315	315	167	169	232	232	176	186	178	178	178	178	296	300	193	199	125	125	403	411	207	251	342	438	198	198
15	c-sz	315	315	167	169	232	232	186	186	178	184	178	178	280	308	187	197	121	121	355	387	227	235	362	366	196	198
16	c-sz	315	317	167	167	216	246	186	186	176	178	178	178	276	292	0	0	121	161	351	351	255	287	270	350	192	198
17	c-sz	315	315	167	169	232	246	176	176	184	184	178	178	304	312	199	199	125	125	351	399	223	223	346	474	198	198
18	c-sz	315	315	169	169	230	246	176	186	178	184	178	178	280	288	193	199	117	125	395	399	219	231	338	474	198	198
19	c-sz	315	315	167	169	232	246	186	186	178	178	178	178	272	284	191	191	129	129	375	391	235	247	0	0	198	198
20	c-sz	315	315	167	167	230	236	176	186	178	178	178	178	264	300	191	199	121	125	347	347	219	239	378	414	198	198
21	c-sz	315	315	167	167	232	232	176	176	184	184	178	178	280	280	193	199	121	125	367	423	235	279	326	474	198	198
22	c-sz	315	317	167	167	222	246	186	186	178	196	0	0	272	292	0	0	125	173	403	403	259	259	274	314	198	198
23	c-sz	315	317	167	171	224	230	164	186	178	178	178	182	292	304	0	0	121	181	383	383	243	243	258	426	180	198
24	c-sz	315	315	167	167	236	246	176	186	178	178	178	182	276	284	191	199	125	125	0	0	0	0	0	0	0	0
25	c-sz	315	315	167	169	232	246	186	186	178	178	178	178	272	300	193	199	125	125	379	387	215	231	362	462	198	198
26	c-sz	315	315	167	167	230	246	176	186	176	180	178	178	292	308	193	193	125	125	363	387	203	207	342	366	198	198
27	c-sz	0	0	167	171	0	0	0	0	0	0	178	178	292	296	191	199	121	125	347	407	235	263	310	434	198	198
28	c-sz	315	315	167	167	246	250	176	186	184	184	178	178	276	280	187	193	125	125	395	399	207	231	450	502	198	198
29	c-sz	315	315	167	167	242	246	176	186	178	184	180	180	292	312	193	199	121	125	375	391	207	215	458	510	198	198
30	c-sz	315	315	167	167	232	246	176	176	178	184	178	178	272	296	187	199	125	129	343	427	203	259	374	470	198	198
31	c-sz	315	315	169	169	236	246	186	186	178	178	178	180	284	296	187	205	125	125	391	391	223	283	346	410	198	198
52	k	315	315	167	167	230	246	176	176	178	178	178	180	276	292	187	193	125	125	359	359	231	239	338	358	198	198
53	k	315	315	169	169	230	236	176	176	178	178	178	178	288	288	193	199	125	125	375	383	207	247	362	494	0	0
54	k	315	315	167	169	230	246	176	176	178	178	178	178	280	280	187	209	121	125	355	415	231	251	310	354	198	198
55	k	315	315	169	171	230	232	176	176	178	184	178	180	288	292	187	209	125	125	355	367	231	247	346	462	198	198
56	k	315	315	167	167	236	246	176	176	176	178	178	178	288	296	193	193	125	125	351	359	255	267	366	486	198	198
57	k	315	315	167	169	232	232	176	176	178	178	178	178	280	292	193	193	125	125	339	383	235	251	330	374	198	198
58	k	315	315	167	167	230	230	176	176	178	178	178	180	276	284	199	199	117	125	331	395	231	243	330	482	198	198
59	k	315	315	169	169	230	230	176	186	178	178	178	178	288	292	193	199	125	129	347	351	215	271	354	354	198	198
60	k	315	315	167	171	230	232	176	176	178	178	178	178	272	272	193	199	125	129	379	387	219	279	398	398	198	198
61	k	315	315	169	169	232	232	176	176	176	178	178	178	276	284	193	199	125	125	363	415	247	267	358	482	198	198
62	k	315	315	167	167	230	236	176	176	178	178	178	178	276	280	199	199	117	129	339	375	231	235	378	482	198	198
63	k	315	315	167	167	230	232	176	176	178	178	178	178	276	280	199	199	125	125	415	427	239	267	358	378	198	198
64	k	315	315	167	169	232	238	176	186	178	178	178	178	272	284	193	199	125	125	331	371	247	267	330	358	198	198
65	k	315	315	167	167	236	246	176	176	178	184	178	178	284	288	193	199	125	125	359	375	215	267	330	366	198	198
66	k	315	315	167	169	232	246	176	176	178	184	178	180	280	280	199	199	117	125	375	383	259	267	346	374	198	198
67	k	315	315	167	167	236	246	176	176	178	178	178	178	276	288	199	199	125	125	339	415	239	247	410	470	198	198
68	k	315	315	167	169	230	232	176	176	184	184	178	178	280	292	193	201	125	125	331	367	215	263	326	458	198	198
69	k	315	315	167	167	232	236	176	176	178	178	178	178	268	312	199	207	125	129	331	339	231	267	346	378	198	198
70	k	315	315	167	167	236	246	176	176	178	178	178	180	284	288	199	207	125	125	383	383	215	227	338	370	198	198
71	k	315	315	167	169	230	246	176	176	178	178	178	178	280	292	199	207	125	125	375	399	231	235	330	370	198	198
72	k	315	315	169	169	230	238	176	176	176	178	178	178	280	312	187	199	125	125	367	387	215	267	470	482	198	198
73	k	315	315	167	167	230	232	176	176	176	178	178	178	268	296	195	199	125	125	367	367	239	267	346	366	198	198
74	k	315	315	167	167	236	246	176	186	178	178	178	178	284	296	193	199	129	129	371	379	215	235	354	374	198	198
75	k	315	315	167	167	236	236	176	176	178	184	178	178	292	312	199	207	125	129	331	367	207	227	370	394	198	198
76	k	315	315	167	167	232	246	176	176	178	184	178	178	280	288	199	199	125	125	383	407	231	247	338	374	198	198
77	k	315	315	167	169	230	230	176	176	178	178	178	178	276	280	199	199	125	125	383	395	215	231	318	370	198	198

78	k	315	315	169	169	230	232	176	176	178	184	178	178	280	280	193	199	125	125	383	387	235	267	330	398	198	198
79	k	315	315	167	167	232	236	176	176	178	178	178	180	280	292	187	195	125	125	383	411	207	271	362	398	198	198
80	k	315	315	167	169	232	236	176	176	178	178	178	178	272	280	193	193	125	129	379	387	235	267	354	462	198	198
155	kt	0	0	0	0	0	0	0	0	0	0	178	178	280	300	193	199	125	125	367	407	259	267	482	578	198	198
156	kt	317	317	167	171	238	254	176	176	164	178	178	178	280	280	187	187	121	125	359	375	259	267	334	478	198	198
157	kt	317	317	167	169	232	232	176	186	164	178	178	178	272	276	199	199	129	129	367	375	271	271	310	398	198	198
158	kt	317	317	167	169	242	254	176	186	164	178	178	180	272	288	193	199	129	129	367	399	0	0	314	370	198	198
159	kt	317	317	169	169	232	234	176	176	164	164	178	178	280	288	187	207	125	125	0	0	267	267	350	426	198	198
160	kt	317	317	167	167	236	236	176	176	164	164	178	178	284	300	187	187	125	125	335	413	247	267	350	354	198	198
161	kt	317	317	167	167	234	234	176	186	164	164	178	184	300	300	193	193	125	129	371	375	247	247	318	422	198	198
162	kt	317	317	167	171	232	232	176	176	164	178	178	178	284	300	191	207	125	129	367	411	255	267	354	474	198	198
163	kt	317	317	167	169	236	236	176	176	164	178	178	178	272	284	193	199	125	125	359	371	243	247	354	382	198	198
164	kt	317	317	167	169	236	236	176	176	164	178	178	178	284	296	193	193	121	125	359	371	239	247	358	370	198	198
165	kt	0	0	167	167	270	270	174	176	164	178	178	178	288	300	193	199	121	125	391	411	223	247	350	394	198	198
166	kt	317	317	167	167	236	236	176	186	164	178	178	178	280	296	199	207	125	129	407	407	219	247	338	478	198	198
167	kt	317	317	167	171	254	254	176	176	164	178	178	178	284	288	187	193	121	129	375	407	239	267	338	418	0	0
168	kt	317	317	167	169	236	238	176	186	164	178	178	182	288	312	193	199	129	129	0	0	0	0	0	0	0	0
169	kt	0	0	167	167	232	234	174	186	164	164	178	178	280	280	187	193	129	129	359	411	215	235	354	386	198	198
170	kt	315	315	167	169	234	238	176	176	164	178	178	178	284	300	187	199	125	129	335	359	267	271	314	334	198	198
171	kt	0	0	0	0	0	0	0	0	0	0	178	178	288	288	193	193	125	125	391	411	267	267	350	398	198	198
172	kt	317	317	169	169	236	252	176	186	164	178	178	178	272	284	193	199	121	129	375	411	259	267	314	350	198	198
173	kt	315	315	167	167	232	248	174	176	164	178	178	178	272	276	187	193	121	121	355	391	275	275	354	478	198	198
174	kt	317	317	167	169	234	236	176	186	164	164	178	180	288	308	185	193	125	125	359	371	235	267	338	526	198	198
175	kt	315	315	167	167	234	252	174	176	164	178	178	178	280	296	187	193	125	125	355	411	267	267	314	522	198	198
176	kt	315	315	169	169	234	236	176	176	164	178	178	178	288	292	193	199	125	129	335	359	247	267	310	358	198	198
177	kt	0	0	167	167	218	232	176	176	164	178	176	180	232	288	0	0	125	201	359	395	247	291	0	0	192	198
178	kt	315	315	167	171	234	238	176	186	164	178	178	178	284	284	187	199	125	125	375	407	247	247	398	398	198	198
179	kt	315	315	167	169	234	234	176	176	164	178	178	178	284	288	193	193	125	129	375	399	259	267	478	482	198	198
180	kt	315	315	167	169	234	252	176	186	164	164	178	180	284	288	193	193	121	125	359	383	227	227	318	354	198	198
181	kt	315	315	167	167	232	248	176	176	164	178	178	178	292	296	193	193	125	125	359	407	227	239	318	478	198	198
182	kt	315	315	167	169	218	236	176	176	164	178	180	180	272	280	185	193	125	129	351	379	247	247	366	478	198	198
183	kt	315	315	167	167	234	234	176	176	164	178	178	178	284	300	189	193	125	125	339	399	267	279	418	470	198	198
184	kt	315	315	167	169	238	242	176	176	164	178	178	180	284	312	185	207	125	129	355	355	239	247	354	354	198	198
185	kt	315	315	167	171	232	234	176	176	164	178	178	178	280	296	193	193	125	125	407	407	267	267	318	414	198	198
186	kt	315	315	167	171	218	238	176	176	164	178	178	180	272	284	187	193	121	125	359	399	239	267	318	402	198	198
187	kt	315	315	169	169	234	236	176	176	164	178	178	182	288	300	193	199	125	125	371	411	239	271	478	478	198	198
312	m	317	317	167	171	252	256	176	176	164	164	178	178	288	288	187	199	125	125	359	383	215	303	366	382	198	198
313	m	317	317	167	171	222	238	178	186	164	164	0	0	0	0	0	0	125	169	351	351	227	227	270	270	180	198
314	m	317	319	169	171	220	242	178	186	164	164	178	180	232	296	0	0	125	165	399	399	291	291	490	490	192	198
315	m	317	317	167	171	238	254	178	186	164	178	178	178	276	292	199	199	125	125	347	375	211	227	434	434	198	198
316	m	317	317	167	171	242	254	178	186	164	178	178	182	288	288	199	199	125	125	419	419	211	267	358	454	198	198
317	m	317	317	167	171	248	248	178	186	164	164	178	178	280	292	199	199	125	125	359	371	231	267	326	442	198	198
318	m	317	317	167	171	238	254	178	186	164	164	172	180	284	304	193	199	125	125	375	395	231	259	426	426	198	198
319	m	0	0	0	0	0	0	0	0	0	0	178	178	276	308	193	193	125	125	375	383	227	227	354	510	198	198
320	m	317	317	167	171	216	256	186	186	164	164	0	0	236	296	0	0	125	181	403	403	227	227	286	454	192	198
321	m	317	319	167	171	220	256	178	186	164	164	178	178	266	304	0	0	125	153	323	323	211	211	222	426	186	198
322	m	315	315	167	171	234	252	176	186	164	164	178	178	280	300	193	199	121	125	359	379	227	227	350	370	198	198
323	m	315	315	167	169	0	0	0	0	164	178	178	178	284	288	193	199	125	125	355	375	203	203	366	450	198	198
324	m	317	317	169	171	234	252	178	186	164	164	178	178	272	288	193	193	121	125	387	395	255	259	362	482	198	198
325	m	317	317	167	171	238	238	178	186	164	164	178	178	276	280	199	199	125	125	383	391	271	271	366	442	198	198
326	m	315	315	167	171	220	242	176	176	164	164	172	178	232	304	0	0	121	165	359	359	227	227	270	310	198	198
327	m	315	317	167	171	222	252	186	186	164	164	172	182	236	300	0	0	125	165	351	351	291	291	318	426	192	198
328	m	317	317	167	171	236	238	176	176	164	164	0	0	284	316	187	199	125	125	351	391	243	243	386	450	198	198
329	m	315	317	167	171	210	236	178	186	164	164	176	178	280	292	0	0	121	189	343	343	227	227	270	510	180	198
330	m	315	315	167	171	234	252	176	176	164	164	178	178	288	288	187	199	125	125	359	415	219	263	374	426	198	198

331	m	0	0	0	0	0	0	0	0	0	0	178	178	276	304	0	0	125	153	399	399	267	267	262	310	180	198
332	m	315	315	167	171	234	242	176	186	164	178	178	178	280	292	187	193	125	125	355	391	235	235	310	374	198	198
333	m	315	315	167	171	242	252	176	176	164	164	172	178	272	304	193	199	125	125	363	403	247	247	470	502	198	198
334	m	315	317	167	171	236	236	176	186	164	164	178	178	296	296	191	193	125	125	383	391	227	275	326	470	198	198
335	m	315	317	167	171	222	236	0	0	164	164	176	178	0	0	0	0	0	0	379	379	227	227	226	314	180	198
336	m	317	317	0	0	220	222	176	186	0	0	0	0	236	236	0	0	173	193	343	343	227	271	258	314	180	180
218	rm	317	317	171	171	248	248	184	186	164	164	176	176	312	324	189	201	117	117	339	379	275	283	358	394	198	198
219	rm	317	317	171	171	246	246	186	186	164	164	176	176	300	300	185	189	117	117	339	367	271	283	358	394	198	198
220	rm	317	317	171	171	246	246	184	184	164	164	172	176	296	296	185	189	117	117	343	367	283	287	378	394	198	198
221	rm	317	317	171	171	246	248	186	186	164	164	172	172	308	312	185	189	117	117	0	0	275	287	434	530	198	198
222	rm	317	317	171	171	246	248	184	186	164	164	178	178	296	296	185	189	117	117	375	387	283	287	342	362	198	198
223	rm	317	317	171	171	246	246	184	186	164	164	176	176	284	300	185	189	117	117	347	399	279	283	362	394	198	198
224	rm	317	317	171	171	246	246	184	186	164	164	176	176	288	300	189	201	117	117	367	387	271	283	378	394	198	198
225	rm	317	317	171	171	246	246	184	186	164	164	0	0	0	0	0	0	0	0	371	375	267	279	358	358	198	198
226	rm	317	317	171	171	246	248	184	186	164	164	172	176	284	300	189	201	117	117	347	387	275	283	358	554	198	198
227	rm	317	317	171	171	246	248	184	186	164	164	172	176	296	316	185	189	117	117	367	383	275	279	378	378	198	198
228	rm	317	317	171	171	240	242	184	186	164	164	176	178	296	304	185	189	117	117	343	375	267	271	358	378	198	198
229	rm	317	317	171	171	246	248	184	186	164	164	176	176	312	316	189	201	117	117	359	387	275	279	378	390	198	198
230	rm	317	317	171	171	246	246	184	186	164	164	176	176	284	316	185	189	117	117	367	399	279	279	378	394	198	198
231	rm	317	317	171	171	240	240	184	184	0	0	176	176	296	300	185	189	117	117	351	379	271	283	378	394	198	198
232	rm	317	317	171	171	246	248	184	186	164	164	176	178	312	316	189	189	117	117	367	387	275	287	390	530	194	198
233	rm	317	317	171	171	246	246	184	186	164	164	172	176	284	284	185	185	117	117	339	387	275	275	394	394	194	198
234	rm	317	317	171	171	246	248	184	186	164	164	176	176	284	316	185	189	117	117	371	379	271	271	358	394	198	198
235	rm	317	317	171	171	246	248	184	184	164	164	176	178	312	316	187	189	117	117	379	387	267	279	358	378	198	198
236	rm	317	317	171	173	246	248	184	186	164	164	172	176	300	324	189	189	117	117	347	367	283	283	358	394	198	198
237	rm	317	317	171	171	240	242	184	186	0	0	172	176	300	316	185	189	117	117	347	351	275	287	362	366	198	198
238	rm	317	317	171	171	246	248	184	184	164	164	172	176	312	316	185	185	117	117	339	379	275	283	378	378	198	198
239	rm	317	317	171	171	248	248	186	186	164	164	176	178	300	300	185	201	117	117	343	387	275	283	362	554	198	198
240	rm	317	317	171	171	246	248	184	186	164	164	172	176	296	296	185	185	117	117	379	387	275	275	378	394	194	198
241	rm	317	317	171	171	246	246	186	186	164	164	178	178	300	316	185	187	117	117	343	379	283	287	358	398	198	198
242	rm	317	317	171	171	240	240	184	186	164	164	176	176	300	316	185	185	117	117	343	359	283	283	354	358	198	198
243	rm	317	317	171	171	246	248	186	186	164	164	176	176	284	316	189	189	117	117	339	367	271	275	358	398	198	198
244	rm	317	317	171	171	240	240	184	186	164	164	178	178	284	312	185	187	117	117	359	379	279	283	358	458	198	198
245	rm	317	317	171	171	248	248	184	186	164	164	176	176	316	316	187	189	117	117	367	379	283	283	362	362	198	198
246	rm	317	317	171	171	246	248	184	186	164	164	176	176	300	312	189	189	117	117	347	359	275	279	378	394	198	198
247	rm	317	317	171	171	240	240	186	186	164	164	176	176	316	316	185	185	117	117	371	387	271	283	394	394	198	198
248	rm	317	317	0	0	246	248	184	186	0	0	176	176	284	316	185	185	117	117	379	387	283	283	362	362	198	198
249	rm	317	317	171	171	246	246	184	186	164	164	176	176	300	300	185	185	117	117	367	379	275	275	394	550	198	198
250	rm	317	317	171	171	246	246	184	186	164	164	176	176	300	300	185	185	117	117	367	379	275	275	394	550	198	198
251	rm	317	317	171	171	246	246	184	184	164	164	176	176	312	316	187	189	117	117	379	383	283	283	358	378	198	198
252	rm	317	317	171	171	246	248	184	186	164	164	176	176	300	300	185	189	117	117	351	371	283	283	354	354	198	198
253	rm	317	317	171	171	246	246	186	186	164	164	176	178	316	316	185	185	117	117	375	379	275	283	378	394	198	198

H_1
GCTGCACGCTACTTTGTAATCTTAGCAGTTATAATACTGATGATGAGTTCCTCGACCTACCTATTTCGTAGGCCCTT
GACCGGAACCTTCCT 24
H_2
GCTGCACGCTACTTTGTAATCCTAGCAGTTATAATACTGACGACGAGTTCCTCAACCTACTTATTTCGTAGGCCCTT
GACCGGAACCTTCCT 68
H_3
GCTGCACGCTACTTTGTAATCCTAGCAGTTATAGTACTGACGACGAGTTCCTCAACCTACTTATTTCGTAGGCCCTT
GACCGGAACCTTCCT 37
H_4
GCTGCACGCTACTTTGTAATCTTAGTAGTTATAATACTGATGATGAGTTCCTCGACCTACCTATTTCGTAGGCCCTT
GACCGGAACCTTCCT 1
H_5
GCTGCACGCTACTTTGTAATTTTAGCAGTTATAATACTGATGATGAGTTCCTCGACCTACCTATTTCGTAGGCCCTT
GACCGGAACCTTCCT 1
H_6
GCCGCACGCCACTTTACAGCCACAGCAGTTACTACGTCACCAGCAAACCCTTCGATCTATCCGACCCGCAGGCCCTT
GACCGGCGCTCTTCC 13
H_7
GCCGCACGCCACTTTACAGCCACAGCAGTTACTACGTCACCAGCAAACCCTTCGATCTATCCGACCCGCAGGCCCTT
GACTGGCGCTCTTCC 1
H_8
GCCGCACGCCACTTTACAGCCACAGCAGTTACTACGTCACCAGCAAACCCTTCGATCTATCCGACCCGCGGGCCCT
TGACCGGCGCTCTTCC 1
H_9
GCCGCACGCCACTTTACAGCCACAGCAGTCACTACGTCACCAGCAAGCCCTTCGATCTATCCGACCCGCAGGCCCT
TGATCGGCGCTCTTCC 1
H_10
GCCGCACGCCACTTTACAGCCACAGCAGTTACTACGTCACCAGCAAACCCTCCGATCTATCCGACCCGCAGGCCCT
TGACCGGCGCTCTTCC 1
H_11
GCCGCACGCCACTTTACAGCCACAGCAGTTACTACGTCACCAACGAGCCTTTCGATCTATCCGACCCGCAGGCCCT
TGATCGGCGCTCTTCC 1
H_12
GCCGCACGCCACTTTACAGCCACAGCAATCACTACGTCACCAGCAAGCCCTTCGATCTATCCGACCCGCAGGCCCT
TGATCGGCGCTCTTCC 1
H_13
GCCGCACGCCACTTTACAGCCACTGCAATTACTACGTCACCAGCAAACCCTTCGATCTATCCGACCCGCAGGCCCTT
GACCGGCGCTCTTCC 1
H_14
GCCGCACGCCACTTTACGGCCACAGCAGTTACTACGTCACCAGCAAACCCTTCGATCTATCCGACCCGCAGGCCCT
TGACCGGCGCTCTTCC 1
H_15
GCCGCACGCCACTTTACAGCCACAGCAGTTACTACGTCACCAGCGAGCCCTTCGATCTATCCGACCCGCAGGCCCT
TGATCGGCGCTCTTCC 1
H_16
GCCGCACGCCACTTTACAGCCACAACAGTTACTACGTCACCAGCAAACCCTTCGTTCTATCCGACCCGCAGGCCCTT
GACCGGCGCTCTTCC 1
H_17
GCCGCACGCCACTTTACGGCCACAGCAGTTACTACGTCACCAGCAAACCCTTCGATCAATCCGACCCGCAAGCCCT
TGACCGGCGCTCTTCC 1
H_18
GCCGCACGCCACTTTACGGCCACAGCAGTTACTACGTCACCAGCAAACCCTTCGATCTTTCCGGCCCGCAAGCCCT
TGACCGGCGCTCTTCC 1
H_19
GCCGCACACCACTTTACGGCCACAGCAGTTACTACGTCACCAGCAAACCCTTCGTTCTATCCGACCCGCAGGCCCTT
GACCGGCGCTCTTCC 1
H_20
GCTGCACGCTACTTTACAGCCACAGCAGTTACTACGTCACCAGCAAACCCTTCAATCTATCCGACCCGCAGGCCCTT
GACCGGGGCTTTTCC 1

H_21
GCTGCACGCTACTTTGTAATCCTAGCAGTTATAATACTGACGACGAGTTCCTCAACCTACTTATTTCGTAGGCCCTT
GCCCGGAACCTTCCT 2
H_22
GCTGCACGCTACATTGTAATCCTAGCAGTTATAATACTGACGACGAGTTCCTCAACCTACTTATTTCGTAGGCCCTT
GACCGGAACCTTCCT 1
H_23
GCTGCACGCTACTTTGTAATCCTAGCAGTTATAATACTGACGACGAGTTCCTCAACCTACTTATTTCGTAGGCCCTT
GACCGGAACCTTCAT 2
H_24
GCTGCACGCTACTTTGTAATCCTAGCAGTTATAGTACTGACGACGAGTTCCTCAACTTACTTATTTCGTAGGCCCTT
GACCGGAACCTTCCT 2
H_25
GCTGCACGCTACTTTGTAATCCTAGCAGGTATAGTACTGACGACGAGTTCCTCAACTTACTTATTTCGTAGGCCCTT
GACCGGAACCTTCCT 1
H_26
GCTGCACGTCACTTTACAGCCACAGCAGTTACTACGTCACCAGCAAACCCTTCGATCTATCCGACCCGCAGGCCCTT
GACCGCGGCTCTTCC 1
H_27
GCTGCACGCTACTTTGTAATCCTAGCAGTTATAGTACTGACGACGAGTTCCTCAACCTACTTATTTCGTAGGCCCTT
GACCGAAACCTTCCT 1
H_28
GCTGCACGCTAGTTTGTAATCCTAGCAGTTATAGTACTGACGACGAGTTCCTCAACCTACTTATTTCGTAGGCCCTT
GACCGGAACCTTCCT 1
H_29
GCCGCACGCCACTTTACAGCCACAGCAGTCACTACGTCACCAGCAAGCCCTTCGATCTATCCGACCCGCAGACCCT
TGATCGGCGCTCTTCC 1
H_30
GCTGCACGCTACTTTGTAATCCTAGCAGTTATAATACTGACGACGAGTTCCTCAACCTACTTATTTCGTAGGCCCTT
GACCGAAACCTTCCT 4
H_31
GCTGCACGCTACTTTGTAATCCTAGCAGTTATAATACTGACGACGAGTTCCTCAACCTACTTATTTCGTAGGCCCTT
GCCCGAAACCTTCCT 1
H_32
GCTGCACGCTACTTTGTAATCCTAGCAGTTATAATACTGACGACGAGTTCCTCAACCTACTTATTTCGTAGGCCCTT
GACCGCAACCTTCCT 1
H_33
GCTGCACGCTACTTTGTAATCCTAGCAGTTATAGTACTGACGACGAGTTCCTCAACCTACTTATTTCGTAGGCCCTT
GCCCGGAACCTTCCT 2
H_34
GCCGCACGCCACTTTACAGCCACAGCAGTTACTACGTCACCAGCAAACCCTTCGATCTATCCGACCCGCAGGCCCTT
GACCGGAACCTTCAA 1
H_35
GCCGCACGCCACTTTACAGCCACAGCAGTTACTACGTCACCAGCAAACCCTTCGATCTATCCGACCCGCAGGCCCTT
GACCGGGGCTCTTCC 1
H_36
GCTGCACGCTACTTTGTAATCCTAGCAGTTATAGTACTGACGACGAGTTCCTCAACCTACTTATTTCGTAGGCACTT
GACCGGAACCTTCCT 1
H_37
CCTGCACGCTACTTTGTAATCCTAGCAGTTATAGTACTGACGACGAGTTCCTCAACCTACTTATTTCGTAGGCCCTT
GACCGGAATCTTCCT 1
H_38
GCTTCACGCTACTTTGTAAACTTAGCCGTTATAATACTGATGATGAGTTCCTCGACCTACCTATTTCGTAGGCCCTT
GACCGGAACCTTCCT 1
H_39
GATAAACACTACTTTGTAAACTTAGCCGTTATAATACTGATGATGAGTTCCTCGACCTACCTATTTCGTAGGCCCTT
GACCGGAACCTACCT 1
H_40
GCTGCACGCTACTTTGTAAGCGTAGCAGTTATAATACTGACGATGAGTTCCTCGACCTACCTATTTCGTAGGCCCT
TGACCCGAACCTTCCT 1
H_41

GCTGCACGCTACTTTGTAAGCGTAGCAGTTATAATACTGACGATGAGTTCCTCGACCTACCTATTTCGTAGGCCCT
TGACCGGAACCTTCCT 1
H_42
GCTGCAAGCTACTTTGTAATCTTAGCAGTTATAATACTGACGATGAGTTCCTCGACCTACCTATTTCGTAGGCCCTT
GACCGGAACCTTCCT 1
H_43
GCTGCACGCTACTTTGTAATCTTAGCAGTTATAATACTGACGATGAGTTCCTCGACCTACCTATTTCGTAGGCCCTT
GACCGGAACCTTCCT 4
H_44
GCTGCACGCTACTTTGTAATCTTAGCAGTTATAATACTGATGATGAGTTCCTCGACCTACCTATTTCGTAGGCCCTT
GACCGGAATCTTCCT 1
H_45
GCTGCACGCTACTTTGTAATCTTAGCAGTTATAATACTGATGATGAGTTCCTTGACCTACCTATTTCGTAGGTCCTT
GACCGGAACCTTCCT 1
H_46
GCTGCACGCTACTCTGTAATCCTAGCAGTTATAGTACTGACGACGAGTTCCTCAACCTACTTATTTCGTAGGCCCTT
GACCGGAACCTTCCT 1
H_47
GCTGCACGCTACTCTGTAATCCTAGCAGTTATAATACTGACGACGAGTTCCTCAACCTACTTATTTCGTAGGCCCTT
GACCGGAACCTTCCT 1
H_48
GCTGCACGCTACTTTGTAATCCTAGCAGTTGTAATACTGACGACGAGTTCCTCAACCTACTTATTTCGTAGGCCCTT
GACCGGAACCTTCCT 2
H_49
GCCGCACGCCACTTTACAGCCACAGCAGTTACTACGTCACCAGCACACCCTTCGATCTATCCGACCCGCAGGCCCTT
GACCGGCGCTCTTCC 1
H_50
GCCGCACGTCACTTTACAGCCACAGCAGTTACTACGTCACCAGCAAACCCTTCGATCTATCCGACCCGCAGGCCCTT
GACCGGCGCTCTTCC 1
H_51
GCTGCTCGCTACTTTGTAATCCTAGCAGTTATAGTACTGACGACGAGTTCCTCAACCTACTTATTTCGTAGGCCCTT
GACCGGAACCTTCCT 1
H_52
GCTGCACGCCACTTTACAGCCACAGCAGTTACTACGTCACCAGCAAACCCTTCGATCTATCCGACCCGCAGGCCCTT
GACCGGCGCTCTTCC 1
H_53
GCCGCACGCCACTTTACAGCCACAGCAGTTACTACGTCACCAGCAAACCCTTCGATCTATCCGACCCGCAGGCCCTT
GACCGGCGCTTTTCC 2
H_54
GCTGCACGCTACTTTGTAATCCTAGCAGTTATAGTACTGACGACGAGTTCCTCAACCTACTTATTTTCTAGGCCTCT
GACCGGAACCTTCCT 1
H_55
GCTGCACGCTGCTTGGTAATCCTAGCAGTTATAATACCGACGACGAGTTCCTCAACCTACTTATTTCGTAGGCCCTT
GACCGGAACCTTCCT 1
H_56
GCTGCACGCCACTTTATAGCCACAGCAGTTACTACGTCACCAGCAAACCCTTCGATCTATCCGACCCGCAGGCCCCC
GACCGGCGCTCTTCC 1
H_57
GCTGCACGCTACTTTGTAAACCTAGCAGTTATAGTACTGACGACGAGTTCCTCAACCTACTTATTTCGTAGGCCTCC
CACCGGAACCTTCCT 1
H_58
GCTGCACCCTACTTTGTAATCCTAGCAGTTATAATACTGACGACGAGTTCCTCAACCTACTTATTTCGTAGGCCCTT
GACCGGAACCTTCCT 1
